# Characterization of Functional Antibody and Memory B-Cell Responses to pH1N1 Monovalent Vaccine in HIV-Infected Children and Youth

**DOI:** 10.1371/journal.pone.0118567

**Published:** 2015-03-18

**Authors:** Donna J. Curtis, Petronella Muresan, Sharon Nachman, Terence Fenton, Kelly M. Richardson, Teresa Dominguez, Patricia M. Flynn, Stephen A. Spector, Coleen K. Cunningham, Anthony Bloom, Adriana Weinberg

**Affiliations:** 1 Pediatric Infectious Diseases, University of Colorado School of Medicine, Aurora, Colorado, United States of America; 2 Statistical and Data Analysis Center, Harvard School of Public Health, Boston, Massachusetts, United States of America; 3 SUNY Stony Brook, Stony Brook, New York, United States of America; 4 St. Jude's Children's Hospital, Memphis, Tennessee, United States of America; 5 University of California San Diego, La Jolla, California, and Rady Children’s Hospital, San Diego, California, United States of America; 6 Duke University Medical Center, Durham, North Carolina, United States of America; 7 Frontier Science and Technology Research Foundation, Amherst, New York, United States of America; 8 Departments of Medicine and Pathology, University of Colorado School of Medicine, Aurora, Colorado, United States of America; Public Health England, UNITED KINGDOM

## Abstract

**Objectives:**

We investigated immune determinants of antibody responses and B-cell memory to pH1N1 vaccine in HIV-infected children.

**Methods:**

Ninety subjects 4 to <25 years of age received two double doses of pH1N1 vaccine. Serum and cells were frozen at baseline, after each vaccination, and at 28 weeks post-immunization. Hemagglutination inhibition (HAI) titers, avidity indices (AI), B-cell subsets, and pH1N1 IgG and IgA antigen secreting cells (ASC) were measured at baseline and after each vaccination. Neutralizing antibodies and pH1N1-specific Th1, Th2 and Tfh cytokines were measured at baseline and post-dose 1.

**Results:**

At entry, 26 (29%) subjects had pH1N1 protective HAI titers (≥1:40). pH1N1-specific HAI, neutralizing titers, AI, IgG ASC, IL-2 and IL-4 increased in response to vaccination (p<0.05), but IgA ASC, IL-5, IL-13, IL-21, IFNγ and B-cell subsets did not change. Subjects with baseline HAI ≥1:40 had significantly greater increases in IgG ASC and AI after immunization compared with those with HAI <1:40. Neutralizing titers and AI after vaccination increased with older age. High pH1N1 HAI responses were associated with increased IgG ASC, IFNγ, IL-2, microneutralizion titers, and AI. Microneutralization titers after vaccination increased with high IgG ASC and IL-2 responses. IgG ASC also increased with high IFNγ responses. CD4% and viral load did not predict the immune responses post-vaccination, but the B-cell distribution did. Notably, vaccine immunogenicity increased with high CD19+CD21+CD27+% resting memory, high CD19+CD10+CD27+% immature activated, low CD19+CD21-CD27-CD20-% tissue-like, low CD19+CD21-CD27-CD20-% transitional and low CD19+CD38+HLADR+% activated B-cell subsets.

**Conclusions:**

HIV-infected children on HAART mount a broad B-cell memory response to pH1N1 vaccine, which was higher for subjects with baseline HAI≥1:40 and increased with age, presumably due to prior exposure to pH1N1 or to other influenza vaccination/infection. The response to the vaccine was dependent on B-cell subset distribution, but not on CD4 counts or viral load.

**Trial Registration:**

ClinicalTrials.gov NCT00992836

## Introduction

Influenza viruses cause yearly epidemics and occasional pandemics that are associated with significant morbidity and mortality. Immunocompromised individuals, including HIV-infected children and adults, have higher rates of influenza morbidity and mortality, proportionate to their degree of immunodeficiency [1–3]. Studies of immune correlates of protection against influenza infection have identified the role of neutralizing antibodies in preventing infection of the host cells and of cell-mediated immunity (CMI) in clearing already-infected cells. Furthermore, hemagglutination inhibition (HAI) antibody titers ≥1:40 were associated with a 50% decrease in the incidence of influenza disease. This observation led HAI titers ≥ 1:40 to become the current benchmark for evaluating the immunogenicity of influenza vaccines.

HIV-infected individuals generally have poor antibody and CMI responses to influenza vaccines, particularly in the context of advanced HIV disease and in the absence of highly active antiretroviral therapy (HAART) [4–6]. Individuals who do not have progressive HIV-1 disease and/or are receiving HAART have improved responses to vaccines [7–9], but do not tend to reach the same HAI titers or CMI as healthy age-matched controls. The mechanisms underlying the poor antibody responses to influenza vaccines in HIV-infected individuals are only partially understood. Antibody responses to influenza vaccines are T-cell dependent and, therefore, are affected by the functionality of T helper 1 (Th1) cells, which play an important role in antibody responses to viral pathogens [10], and of T follicular helper (Tfh) cells, which have recently been identified as the key stimulators of T-dependent antibody production [11]. Both Th1 and Tfh functions are compromised in HIV-infected individuals, contributing to the low immunogenicity of vaccines including influenza [12–14]. In addition, multiple B-cell abnormalities have been identified in HIV-infected individuals [15], which may also play a role in the poor antibody responses to vaccines. Although HIV does not replicate in B cells, it interferes with B-cell function through multiple interactions: gp120 and cellular DC-SIGN; CD40L incorporated into the virion membrane and cellular CD40; and complement fixing HIV antigen-antibody complexes with cellular CD21 [16–22]. In addition, HIV Nef protein can be delivered to the B cells through immunologic synapses with CD4+ T cells and/or macrophages and impede the NFkB pathway, while also activating the SOCS pathway [19]. Additional indirect effects of HIV on B cells result from inflammation and lymphopenia. These ultimately translate into impaired immunoglobulin class switch recombination, loss of resting memory B cells (CD21+CD27+), abnormally high proportions of immature (CD10+) and activated (CD21-CD27+, CD95+ and/or CD38+) B cells, and increased B-cell turn-over and apoptosis [19,23–25]. All these factors may contribute to the decreased antibody responses to infections and vaccines [18,26–29]. Furthermore, only some of the B-cell abnormalities are averted by lack of disease progression or reversed with HAART [30,31]. To best target the efforts to improve the immunogenicity of vaccines in HIV-infected individuals, it is critically important to understand the relative contributions of each of these factors to the B cell responses to vaccines.

The goal of this study was to assess the effect of abnormal B-cell maturation and activation and/or dysfunctional T-cell help on the magnitude, quality and memory of the humoral immune response to a pH1N1 vaccine. The primary outcome measures of this analysis were HAI and microneutralization titers, antibody avidity, and B cell memory measured by ELISPOT. The subjects were HIV-infected children and youth who participated in the International Maternal Pediatric and Adolescent Clinical Trials (IMPAACT) Network’s P1088, a study of the safety and immunogenicity of pH1N1 monovalent influenza immunization [32]. P1088 HAI results and analysis of the regulatory B and T cells have been previously reported by Flynn *et al* [32] and Weinberg *et al* [33] respectively. Here, we provide a detailed analysis of neutralizing antibody responses, antibody avidity and IgG and IgA B-cell memory as a function of Th1, Tfh, circulating B-cell subsets, age and HIV disease characteristics.

## Subjects and Methods

### Study Design

This was a substudy of P1088 (NCT00992836), approved through an amendment to the parent P1088 study entitled New Works Concept Sheet 114 (NWCS 114). In the parent study, 155 perinatally HIV-infected children and youth, aged ≥4 to <25 years, were recruited from 37 different IMPAACT sites in the US and Puerto Rico between October 14 and November 12, 2009. Subjects on stable antiretroviral therapy for ≥90 days prior to entry received two 30 μg doses of 2009 Novartis Influenza A (pH1N1) monovalent vaccine (Fluvirin), separated by 21–28 days. Seasonal influenza vaccine administration was restricted to ≥2 weeks before enrollment and to ≥2 weeks after the 2^nd^ pH1N1 dose. A full description of the parent study can be found in Flynn *et al*, JID 206:421–430.[32]

**Fig 1:**
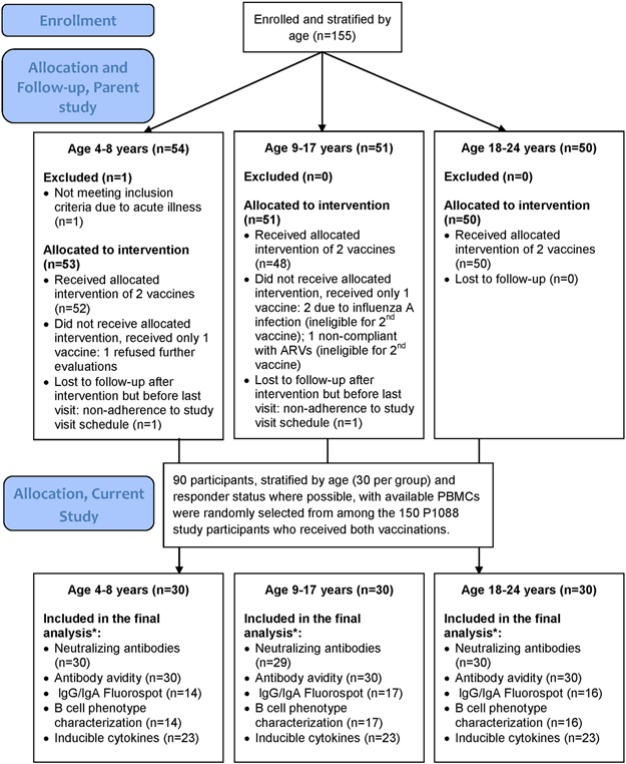
Flow Chart after Enrollment. Abbreviations: ARVs, antiretrovirals; HAI, hemagglutinin inhibition; PBMCs, Peripheral blood mononuclear cells. *Reasons for exclusion from the final analysis included: insufficient sample to perform assay, invalid results of assay

The enrollment in the parent study was stratified into 3 age groups (Fig. 1), chosen based on immunologic and physiologic differences. Children <9 years of age generally mount insufficient antibody responses to influenza vaccines administered for the first time. This is generally accepted and acknowledged by the CDC recommendation to administer 2 doses of vaccine under these circumstances. The age of 18 years is generally considered the transition from adolescence to adulthood, which may also be associated with immunologic changes. Therefore the accrual was stratified into 3 age groups: 4 to <9, 9 to <18 and 18 to <25 years of age. The current substudy followed the same stratification for subject inclusion and analysis. 90 participants with available samples were selected from the 155 P1088 study participants, such that both complete responders (defined by an HAI titer ≥1:40 and ≥ 4-fold increase post-dose 1 compared to baseline) and non-responders were well represented, with the goal of having up to half of the subjects in each group be non-responders. This required including all nonresponders with available samples and randomly selecting responders to complete the N = 30 for each age group.

Vaccines were given at week 0 (baseline) and at 21–28 days after the first vaccine. Blood for immunologic assays was collected at week 0, 21–28 days post-dose 1 (coinciding with the administration of the 2^nd^ vaccine), 10–14 days post-dose 2, and at 28 weeks post-dose 1. The timing of the blood draws was designed to obtain a pre-vaccination sample, a sample at the peak of the immune response to vaccine dose 1 (and to allow at least 3–4 weeks between vaccinations), a sample at the peak of the immune response to dose 2, and a sample at the end of the influenza season to measure persistence of the antibodies. Throughout the manuscript, these timepoints are referred to as: baseline or week 0, post-dose 1 or week 3, post-dose 2 or week 5, and 28 weeks.

The CONSORT Checklist, the original P1088 protocol for this trial, and the P1088 Protocol and P1088 NWCS 114 Amendment for this current study are available as supporting information (see S1 CONSORT Checklist, S1 Protocol, S2 Protocol). The study was approved by local IRBs. Written consent and assent were obtained from participants and/or legal guardians as appropriate. Further information on the participating sites and their Institutional Review Boards can be found in the Acknowledgments and (see S1 List for a list of participating IMPAACT sites). This substudy was approved by the Colorado Multiple Institutional Review Board (COMIRB), and all assays of the substudy were performed at the University of Colorado School of Medicine.

### Neutralizing antibodies

An ELISA-based microneutralization was performed as previously described [34,35] on sera collected at baseline and post-dose 1. Briefly, serial 2-fold dilutions of sera starting at 1:10 in phosphate-buffered solution (PBS) were added in duplicate to 100 TCID_50_ of pH1N1 influenza virus and 1.5 x 10^4^ MDCK cells in 96-well microtiter plates. Cell control and virus control wells were included on each plate. After 18–22 hours of incubation in a humidified atmosphere at 37°C and 5% CO_2_, the cell monolayer was fixed and the ELISA was performed using 100μL anti-influenza A NP mouse antibodies (Millipore, cat. # MAB8257 & MAB8258) followed by goat anti-mouse IgG conjugated to horseradish peroxidase (HRP) secondary antibodies (KPL, cat. # 074–1802). Bound antibodies were revealed with 3,3’,5,5’-Tetramethylbenzidine (TMB) substrate. The optical density (OD) was measured with a Multiscan FC ELISA reader (Thermo Fisher) using a 450 nm filter. The neutralizing titer was calculated using the following equation: [median OD of virus control wells + median OD of cell control wells]/2. Samples with discordance larger than 1 dilution between replicates were repeated.

### Antibody avidity

The assay was adapted from a previously described ELISA-based method with urea denaturation [36]. 96-well Immulon 2 HB microplates (Thermo Scientific) were coated for 20–24 hours at 4°C[36] with monovalent 2009 pH1N1 inactivated influenza vaccine (Sanofi-Pasteur) at a pre-optimized amount of 15ng/well. After blocking (PBS with 0.1% Tween and 0.2% FBS) for 120 min, 100μL of serum diluted at 1:1000 in PBS were added to duplicate wells. Plates were incubated with serum for 1 hour at 37°C after which 100μL of 8M urea solution in PBS were added to urea-treated wells and 100μL of PBS to control wells. After a 15 minute-incubation at room temperature, plates were washed and bound antibodies were revealed with HRP-conjugated goat anti-human IgG antibodies (Sigma-Aldrich; A0170) and TMB substrate as above. Titers were calculated by interpolating the average OD of replicate test wells on a standard curve created with serial dilutions of a unique control sample. Results were considered valid if the OD of the test sample was within the dynamic range of the assay. For OD’s outside the dynamic range, the test was repeated at a higher or lower dilution. The avidity index (AI) was calculated by dividing the ELISA units of the urea-treated sample by those of the urea-untreated sample.

### IgG/IgA FluoroSpot

Cryopreserved PBMCs were used for the detection of IgG/IgA antibody secreting cells (ASC). Cells were thawed, counted and then stimulated in RBMI 1640 (Gibco) with 10% fetal bovine serum (Gemini Bio-Products), 0.4% penicillin and streptomycin, 1% Hepes buffer (Corning Cellgrow), 1μg/mL *Staphylococcus aureus* Cowan (Sigma-Aldrich), 6μg/mL CpG (Operon Technologies), and 1μg/mL pokeweed mitogen (Sigma-Aldrich) for 96 hours at 37° C, 5% CO_2_. PBMCs with viability ≥55% were used at 50,000 cells/well in duplicate wells of the FluoroSpot IgG/IgA kits (Mabtech Inc.). Assays were performed as per manufacturer’s instructions using pH1N1 antigen, HAI titer 1:1024, to coat the plates and an ImmunoSpot II (Cell Technologies Ltd.) instrument to count the ASC. Results are expressed as ASC/10^6^ stimulated PBMCs.

### B-cell phenotypic characterization

B-cell subsets were measured in freshly thawed cryopreserved PBMCs. After washing and counting viable cells, PBMCs were surface-stained with the following conjugated mAbs: anti-CD19-PE-Cy5, anti-CD21-PE, anti-CD20-PE-Cy7, anti-CD27-APC, anti-IgM-FITC, anti-CD38-FITC, anti-HLA-DR-PE-Cy7 (BD Bioscience); anti-CD10-APC-Cy7, anti-BAFFr-APC-Cy7 (Biolegend); anti-TACI-PE, anti-CxCr5-APC (R & D Systems) and analyzed with Guava easyCyte 8HT (Millipore) and FlowJo (Treestar). Subsets were expressed as percentages of the parent CD19+ B-cell population.

### Inducible cytokines

IFNγ, IL-2, IL-4, IL-5, and IL-13 were measured by multiplex bead array and IL-21 by ELISA using supernatants of PBMC cultures stimulated for 48 hours with pH1N1 and mock control as previously described [33]. Culture supernatants were stored at -80°C until assayed in batch. The bead array assays used the MILLIPLEX MAP High Sensitivity Human Cytokine Magnetic Bead Panel kit (Millipore; HSCYTMAG-60SK) on the Bio-Rad Bio-Plex 200 instrument per manufacturer’s instructions. The lower level of detection (LLOD) was 0.08–1.01pg/mL and the dynamic range 13–2,000pg/mL. Data were analyzed using Bio-Plex manager 5.0 software (manufacturer Bio Rad) and concentrations were interpolated on the manufacturer’s standard curve using PRISM software (Graphpad). IL-21 concentrations were measured with the Human IL-21 ELISA Ready-SET-Go! (e-Bioscience; 88–7216) kit per manufacturer’s protocol. The LLOD was 31 pg/mL and the dynamic range 31–4,000pg/mL. OD’s were measured as above. Test supernatant concentrations were calculated using PRISM software.

### Statistical Analyses

The baseline HIV disease characteristics were summarized and compared among the three age groups using a Fisher’s Exact test for gender, race (black vs. other), ethnicity (Latino vs. other) and an ANOVA test for CD4%, CD8% and log_10_ RNA count. The rates of HAI and microneutralization titers ≥1:40 at baseline were compared among the three age groups using a Fisher’s Exact test. HAI and microneutralization titers <1:10 were considered undetectable and were assigned a value of 1:5. Microneutralization titers post vaccination and pre-post changes in these titers were compared across the three age groups using a Kruskal-Wallis test. Changes in antibody avidity, B-cell memory responses, phenotypic characterization of B-cell subsets and inducible cytokine levels between different time points were assessed using Wilcoxon Matched Pairs Signed-Rank tests. The groups of participants with baseline HAI titers ≥40 vs. <40 were compared with respect to these measures post vaccination using a Wilcoxon Rank-Sum test. The final flow cytometry analyses were restricted to samples with ≥40 events in the anchor gate. However, a sensitivity analysis showed that the inclusion of samples with <40 events in the anchor gate would not have changed the results. Spearman correlation analyses were performed to assess the strength of associations on data at specific time points and test their statistical significance. All analyses were performed using SAS Version 9.2 (SAS Institute INC, Cary, NC) and the graphs were produced using the R software.

## Results

### Baseline characteristics of the study population and the HAI responses to pH1N1 vaccine

The characteristics of the 90 children and youth who participated in this substudy are summarized in Table 1. The CD4+ T cells had mean ±S.D. of 33 ±9.3% for the entire cohort and differed significantly across the age groups (the older age group had lower mean than the younger groups, p<0.001). HIV plasma RNA had mean±S.D. of 2.1±0.9 log_10_ copies/mL for the entire cohort and was higher in the older compared with the younger groups (p<0.001). At baseline, 26 participants (29%) were seroprotected against pH1N1 (as defined by HAI titers ≥1:40). Seroprotection at baseline was less common in the younger group (age 4 to <9, 13%) compared with the older groups (age 9 to <18, 30%, and age 18 to <25, 43%). After the 1^st^ dose of vaccine, the proportions of seroprotected subjects and of complete responders (defined by HAI≥1:40 and ≥4-fold increase in antibody titers compared to baseline) were 62/90 (69%) and 56/90 (62%), respectively, and did not significantly differ across age groups (p = 0.41 and 0.80, respectively). Demographic and HIV disease characteristics of the 90 substudy participants and of the parent study participants not included in the substudy were similar (S1 Table).

**Table 1:** Characteristics of the Study Population.

Variable	All	4-<9 years of age	9-<18 years of age	18-<25 years of age	P value among groups^c^
Total Number of Subjects	90	30	30	30	
Gender Female	47 (52%)	16 (53%)	14 (47%)	17 (57%)	0.81*
Race and Ethnicity					
Black	53 (59%)	16 (53%)	18 (60%)	19 (63%)	0.82*
Latino	33 (37%)	15 (50%)	11 (37%)	7 (23%)	0.10*
Age (yrs)					
Mean	13	6	14	20	
Standard Deviation	5.9	1.5	2	1.7	
CD4 Percent					
Mean	33	38	33	28	<0.001**
Standard Deviation	9.3	6.4	7.6	10.7	
CD8 Percent					
Mean	37	28	38	46	<0.001**
Standard Deviation	13.6	6.2	13.9	13.2	
Log 10 RNA Count					
Mean^a^	2.1	1.8	1.99	2.5	0.002**
Standard Deviation	0.9	0.5	0.7	1.1	
Baseline pH1N1 HAI ≥1:40	26 (29%)	4 (13%)	9 (30%)	13 (43%)	0.04*
Post-dose 1 pH1N1 HAI ≥1:40	62 (69%)	18 (60%)	21 (70%)	23 (77%)	0.41*
Post-dose 1 complete response^b^	56 (62%)	17 (57%)	20 (67%)	19 (63%)	0.80*

a. The lower limit of detection varied among subjects depending on the assay used at the specific clinical research site; RNA values below the limit of detection were replaced with the lower limit of the assay (eg, 18 cp/mL was replaced with 50 cp/mL for an assay with the limit of detection of 50 cp/mL).

b. Complete response is defined as persons achieving both seroresponse and seroprotection.

c. P values among age groups were calculated by:

* Fisher’s Exact Test

**Analysis of Variance

### Viral neutralization function of the antibody response to pH1N1 vaccine

Microneutralization assays were performed on all subjects at baseline and post-dose 1 (Table 2). There was a significant increase in the median microneutralization titers in response to vaccination from 1:5 to 1:20 (p<0.0001). Although median titers increased in all age groups from baseline to post-dose 1, the proportion of subjects seroprotected (defined by a titer ≥1:40) after vaccination increased with the ages of the groups (23.3%, 50.0%, and 70.0% for younger, middle and older groups, respectively; p = 0.001). Excluding those with baseline microneutralization titers ≥1:40, the proportions of seroprotected post-vaccination were 17.9%, 38.1%, and 60.9% in the younger, middle and older groups, respectively (p = 0.008). Furthermore, the proportions of subjects who had a ≥4-fold increase in microneutralization titer in response to vaccine also increased with age (36.7%, 44.4% and 70.0%, respectively; p = 0.03). The difference across age groups in the proportion of participants with ≥4-fold-rises was significant among subjects with microneutralization titers <1:40 at baseline (p = 0.03), but not in subjects with baseline titers ≥1:40 (p = 0.3), where the relatively small sample size provided limited precision and power. The proportions of complete responders increased with the ages of the groups regardless of the baseline microneutralization titers.

**Table 2:** Neutralizing Antibody Responses after the 1^st^ Dose of pH1N1 Vaccine.

	Age Groups				
	All (N = 89)	4 to <9 (N = 30)	9 to <18 (N = 29)	18 to <25 (N = 30)	P value among groups^c^
Baseline titer, Median (IQR^a^)	5 (5,5) ^**b**^	5 (5,5)	5 (5,30)	5 (5,20)	0.09*
Post vaccine titer, Median (IQR)	20 (5,80)	10 (5,20)	30 (8,120)	60 (20,160)	0.006*
Median fold-change in titer (IQR) post vaccine	4 (1,8)	2 (1,4)	2 (1,8)	6 (2,16)	0.02*
**% Seroprotection (microneutralization titer ≥1:40)**					
Baseline, all subjects	18.2%	6.7%	25%	23.3%	0.12**
Post vaccine, all subjects	47.7%	23.3%	50%	70%	**0.001** **
Post vaccine, subjects with baseline microneutralization titers <1:40	37.5% (27/72)	17.9% (5/28)	38.1% (8/21)	60.9% (14/23)	**0.008** **
**% Seroresponse (≥4-fold change in titer vs. baseline)**					
All subjects	50.6%	36.7%	44.4%	70%	**0.03** **
Subjects with baseline microneutralization <1:40	48.6% (35/72)	32.1% (9/28)	47.6% (10/21)	69.6% (16/23)	**0.03** **
Subjects with baseline microneutralization ≥1:40	60% (9/15)	100% (2/2)	33.3% (2/6)	71.4% (5/7)	0.3**
**% Complete response (titer ≥1:40 post-vaccine and ≥4-fold change in titer vs. baseline)**					
All subjects	37.1%	23.3%	27.6%	60%	**0.01** **
Subjects with baseline microneutralization <1:40	33.3%	17.9%	28.6%	56.5%	**0.01** **

a. IQR = Interquartile Range

b. Numbers represent the reciprocal of pH1N1 microneutralization titers.

c. P values among age groups were calculated by:

* Kruskall-Wallis Test

**Fisher’s Exact Test

### Avidity of antibody responses to pH1N1 vaccine

**Fig 2:**
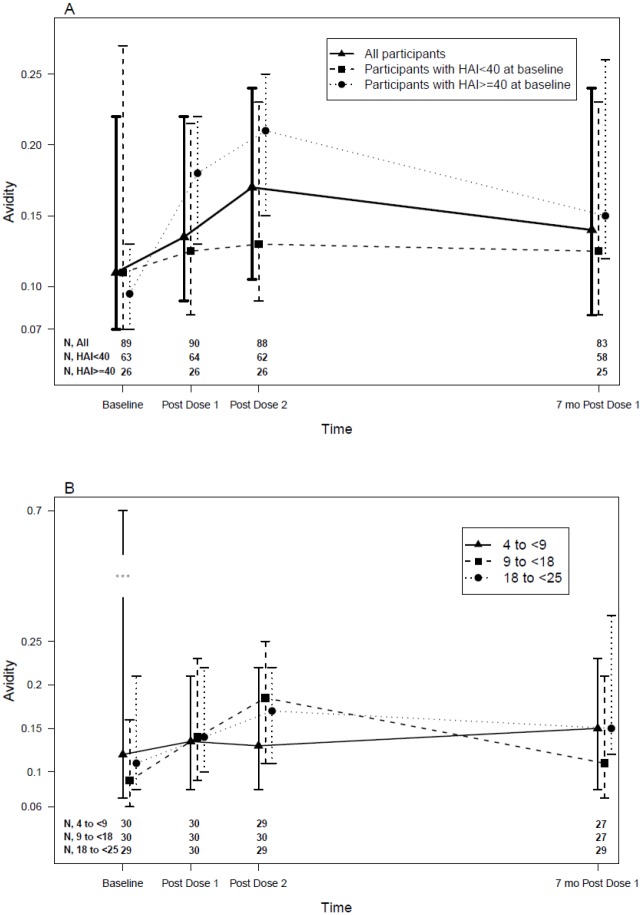
Changes in Antibody Avidity after pH1N1 Vaccination by Age Group and Baseline HAI. Data were derived from HIV-infected children and youth who received 2 doses of pH1N1 monovalent vaccine (30 μg/dose) approximately 3 weeks apart. Data represent medians and Inter Quartile Range (IQR) of the avidity index (AI). The number of subjects per group at each time point is listed on the graph. **A** presents the AI responses for all subjects and also for the subjects divided by baseline pH1N1 HAI immune serostatus (HAI titer <1:40 versus ≥1:40). Overall, AI significantly increased post-dose 2 (p = 0.04) and remained slightly higher at 7 mo (p = 0.07). Only subjects seroprotected at baseline had significant AI increases after vaccination (p<0.0001, <0.0001 and 0.003 post-dose 1, post-dose 2 and 7 months post-dose 1, respectively). Baseline seropositive participants with HAI titers ≥ 1:40 had a significantly higher AI response to vaccination compared with participants with baseline HAI titers <1:40 (p = 0.02 at both post-dose 1 and post-dose 2). **B** shows AI responses in each age group. In the 4 to <9-year old group, there were no significant differences in AI between baseline and any subsequent time point. In the 9 to <18-year old group, there were significant AI increases at post-dose 2 and at 7 months (p = 0.04 and 0.048 respectively). In the 18 to <25-years old group, there were significant differences from baseline at all time points (p = 0.02, 0.04 and 0.002, respectively).

Antibody avidity was measured at baseline, after each dose of vaccine and at 28 weeks. The AI significantly increased (p = 0.04) only after the 2^nd^ dose of vaccine, from a median of 0.11 at baseline to 0.17 post-dose 2 (Fig. 2A) and remained marginally higher than baseline at 0.14 at week 28 (p = 0.07; Fig. 2A). It is interesting to note that only participants with baseline HAI titers ≥1:40 had significant AI increases after vaccination (median = 0.10, 0.18, 0.21 and 0.15 for baseline, post-dose 1, post-dose 2 and week 28 respectively, p of <0.0001, <0.0001 and 0.003 for change from baseline; Fig. 2A), whereas in participants with baseline HAI <1:40 the AI did not change significantly (median = 0.11, 0.13, 0.13 and 0.13 for baseline, post-dose 1, post-dose 2 and week 28 respectively, p≥0.87 for change from baseline). Baseline seropositive participants with HAI titers ≥ 1:40 had a significantly higher AI response to vaccination compared with participants with baseline HAI titers <1:40 (p = 0.02 at both post-dose 1 and post-dose 2; Fig. 2A). AI did not increase after vaccination in the younger age group (median = 0.12, 0.14, 0.13 and 0.15 for baseline, post-dose 1, post-dose 2 and week 28, respectively, p of 0.41, 0.62 and 0.23 for change from baseline; Fig. 2B); AI significantly increased after the 2^nd^ dose of vaccine and remained increased at week 28 in the middle age group (median = 0.09, 0.14, 0.19 and 0.11 for baseline, post-dose 1, post-dose 2 and week 28 respectively, p of 0.2, 0.04 and 0.048 for change from baseline; Fig. 2B); and AI significantly increased at all visits after the 1^st^ dose of vaccine in the older age group (median = 0.11, 0.14, 0.17 and 0.15 for baseline, post-dose 1, post-dose 2 and week 28 respectively, p of 0.02, 0.04 and 0.002 for change from baseline; Fig. 2B).

### B-cell memory responses to pH1N1 vaccine

**Fig 3:**
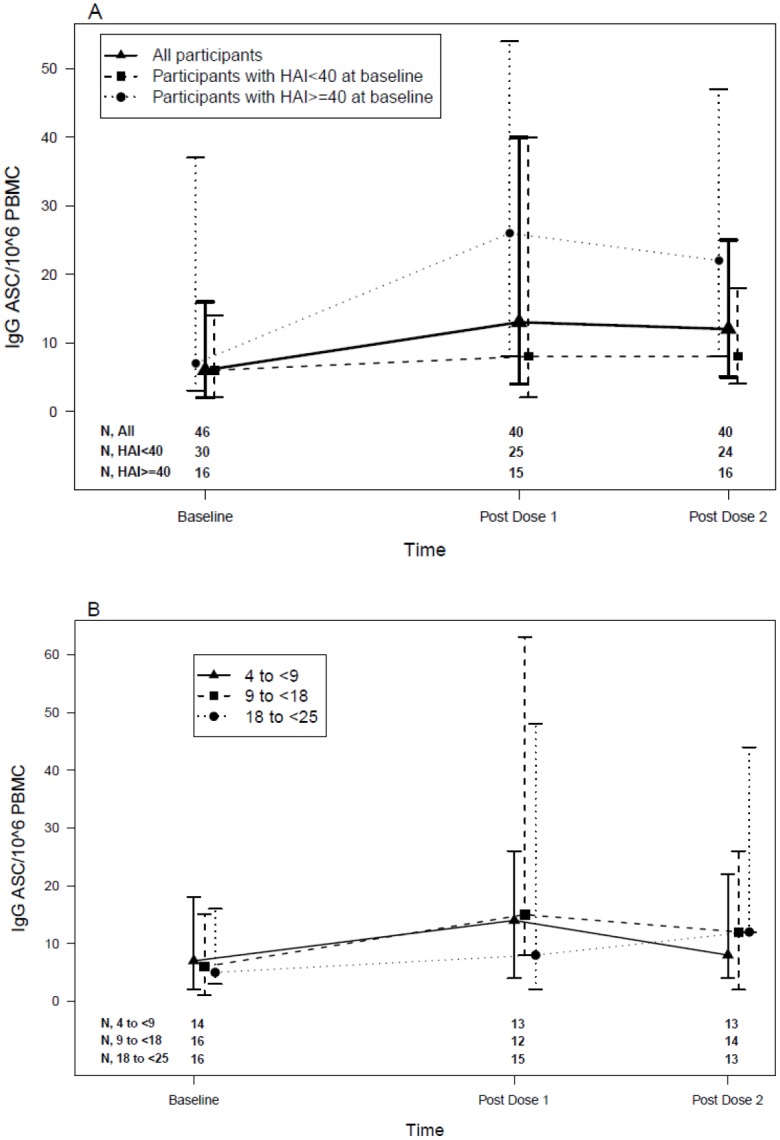
IgG ASC measured by ELISPOT (memory B-cells) at Baseline and after Vaccination. Data were derived from HIV-infected youth and children who received 2 doses of pH1N1 inactivated monovalent vaccine (30 μg/dose) approximately 3 weeks apart. Data represent medians and IQR of the IgG ASC/10^6^ PBMCs at each timepoint. The number of subjects per group at each time point is listed on the graph. **A** presents the IgG responses for all subjects and also for the subjects divided by baseline pH1N1 HAI immune serostatus (HAI titer <1:40 versus ≥1:40). After the first immunization there was a significant increase in IgG ASC (p = 0.048). There were no statistical differences in IgG ASC within each HAI immune serostatus group between baseline and the two vaccinations, but subjects with baseline HAI ≥1:40 titers had significantly higher IgG ASC after vaccination than those with titers <1:40 (p = 0.03). **B** depicts the median IgG ASC by age group. There were no statistical differences in IgG ASC within each age group between baseline and the two vaccinations.

B-cell memory was assessed by the number of pH1N1-specific IgG and IgA antibody secreting cells (ASC)/10^6^ PBMCs. At baseline, the median IgA and IgG ASC were 0 and 6 respectively. After immunization, there was no change in IgA ASC (not depicted), but the IgG ASC significantly increased after the 1^st^ dose to 13 (p = 0.048) and plateaued thereafter (Fig. 3A). Although the analysis of IgG ASC change from baseline post-dose 1 and post-dose 2 did not show significant differences when the groups were stratified by baseline HAI positivity, baseline seropositive participants (HAI titers ≥ 1:40) had significantly higher IgG ASC post-dose 2 compared with baseline seronegative participants (HAI titers <1:40; p = 0.03; Fig. 3A). There were no significant changes in IgG ASC after vaccination when subjects were stratified by age (Fig. 3B).

### pH1N1-specific Th1, Th2 and Tfh responses to pH1N1 vaccine

Th1 cytokines IFNγ and IL-2, Th2 cytokines IL-4, IL-5 and IL-13, and the Tfh cytokine IL-21 were measured at baseline and post-dose 1 in pH1N1-stimulated PBMCs culture supernatants. pH1N1-induced IL-2 and IL-4 were the only cytokines with significant increases from baseline to post-dose 1 (p = 0.01 and p = 0.004 respectively; Table 3).

**Table 3:** Th1, Th2 and Tfh Responses to pH1N1 Vaccine.

Variable^a^	Baseline		Post-dose 1		
	Median (IQR) pg/mL	N	Median (IQR) pg/mL	N	P value^d^
IFNγ^b^	19.69 (4.85,103.67)	71	26.53 (10.89,145.48)	72	0.12
IL-2^b^	56.72 (24.29,105.03)	72	63.38 (35.50,140.01)	72	**0.01**
IL-4^b^	28.70 (9.56,83.32)	67	52.66 (19.02,108.07)	70	**0.004**
IL-5^b^	1.00 (1.00,6.40)	56	1.00 (1.00,5.98)	62	0.78
IL-13^b^	15.81 (8.32,39.30)	72	15.83 (7.36,32.98)	70	0.44
IL-21^c^	75.69 (65.01,84.30)	68	70.80 (54.44,90.93)	70	0.81

a. Th1 (IFNγ, IL-2), Th2 (IL-4, IL-5, IL-13) and Tfh (IL-21) responses were measured in the supernatants of PBMC cultures stimulated with influenza pH1N1 virus.

b. Measured by multiplex bead array (Luminex). Lower levels of detection were 0.08–1.01 pg/mL.

c. Measured by ELISA. Lower level of detection was 31 pg/mL.

d. P values calculated by Wilcoxon Matched Pairs Signed Ranks test.

### Correlations among the pH1N1-specific B- and T-cell immune responses to pH1N1 vaccine

To determine the extent of the coordination between the different components of the immune response to pH1N1 vaccine, including magnitude and functionality of the antibodies and generation of B cell memory and of T-cell help, we performed correlation analyses before and after vaccination between HAI, microneutralization, avidity, IgG ASC and cytokine production (Table 4). Correlation results represent the correlation of the laboratory results at that time point for each of the variables. The data showed strong positive correlations between HAI and microneutralization titers at all time-points (p<0.0001). HAI titers were also significantly correlated with pH1N1-specific IL-2 and IFNg production at all time-points analyzed (p<0.03); with IgG B cell memory responses only after vaccination; and with AI at post-dose 2, when significant AI increases over baseline were detected for the first time. It is important to note that before vaccination high HAI titers were associated with low AI, suggesting that not all HAI antibodies at baseline were specific for pH1N1. The correlation analysis of microneutralization titers followed the same pattern as HAI. AI weakly correlated with IgG ASC at post-dose 1. There were significant correlations of IgG ASC with pH1N1 IFNg production at all time-points analyzed but not with IL-2. pH1N1-specific IL-21 production did not correlate with any of the B cell responses to the vaccine (data not shown).

**Table 4:** Correlations of cellular and humoral B- and T-cell mediated immune responses to pH1N1.

Variable 1	Variable 2	Time point at which data from each variable was analyzed					
		Baseline		Post-dose 1		Post-dose 2	
		ρ (p value)	N	ρ (p value)	N	ρ (p value)	N
pH1N1 HAI Titers	pH1N1 IgG ASC	0.15 (0.32)	46	**0.45 (0.004)**	40	**0.34 (0.03)**	40
	pH1N1 IFNγ	**0.37 (0.002)**	71	**0.26 (0.03)**	72	NM	
	pH1N1 IL-2	**0.31 (0.008)**	72	**0.40 (0.001)**	72	NM	
	Microneutralization Titers	**0.70 (<0.0001)**	88	**0.63 (<0.0001)**	88	NM	
	Antibody Avidity	**-0.22 (0.04)**	89	0.13 (0.21)	90	**0.28 (0.01)**	88
Microneutralization Titers	pH1N1 IgG ASC	0.23 (0.14)	44	**0.45 (0.005)**	38	NM	
	pH1N1 IFNγ	**0.30 (0.01)**	68	0.13 (0.29)	69	NM	
	pH1N1 IL-2	**0.30 (0.01)**	69	**0.39 (0.001)**	69	NM	
	Antibody Avidity	**-0.32 (0.002)**	87	0.14 (0.19)	88	NM	
Antibody Avidity	pH1N1 IgG ASC	-0.03 (0.84)	44	*0*.*29 (0*.*08)*	38	0.09 (0.57)	38
	pH1N1 IFNγ	-0.13 (0.28)	69	0.08 (0.52)	71	NM	
	pH1N1 IL-2	-0.03 (0.81)	70	0.04 (0.75)	71	NM	
B-cell memory (pH1N1 IgG ASC)	pH1N1 IFNγ	**0.39 (0.01)**	43	**0.53 (0.001)**	38	NM	
	pH1N1 IL-2	-0.01 (0.93)	44	0.26 (0.12)	38	NM	

NM = Not measured

### Correlations of the response to pH1N1 vaccine with HIV disease characteristics

Correlation analyses of CD4% and HIV plasma RNA with HAI titers, microneutralization titers, AI and IgG ASC responses to vaccination failed to show any significant associations (S2 Table).

### Circulating B-cell subsets and their relationship with functional B-cell responses to pH1N1 vaccine

The following B cell subsets were enumerated at baseline and after each vaccination: CD19+CD21+CD27+ (resting memory); CD19+CD21+CD27-(naïve); CD19+CD21-CD27-CD20+ (tissue-like memory); CD19+CD21-CD27-CD20- (transitional); CD19+CD21-CD27+CD20- (plasmablasts); CD19+CD10+ (immature); CD19+CD10+CD27+ (immature activated); CD19+HLADR+CD38+ (activated); CD19+BAFFR+; CD19+TACI+; and CD19+CXCR5+. There were no appreciable changes in B-cell subset distribution across visits. (S3 Table).

Several B-cell subsets were significantly associated with the immunogenicity of the vaccine in the HIV-infected study population (Table 5). High CD19+CD10+CD27+% (immature activated) cells and CD19+CD10+% (immature) cells showed a marginal correlation with high pH1N1 HAI post-dose 2 (ρ≥0.26, p≤0.08). An increase in AI was significantly correlated with low CD19+CD21-CD27-CD20+% (tissue-like memory) and CD19+CD21-CD27-CD20-% (transitional) cells post-dose 2 (ρ≤-0.32, p = 0.03) and with high CD19+CD21+CD27+% (resting memory; ρ = 0.29, p = 0.05).

**Table 5:** Correlations of cellular and humoral B- and T-cell mediated immune responses to B-Cell Subsets.

Variable 1	Variable 2	Time point at which data from each variable was analyzed			
		Post-dose 1		Post-dose 2	
		ρ (p value)	N	ρ (p value)	N
pH1N1 HAI Titers	CD19+CD10+CD27+ (immature activated)	-0.09 (0.53)	51	*0*.*28 (0*.*05)*	48
	CD19+CD10+ (immature)	-0.10 (0.48)	51	*0*.*26 (0*.*08)*	48
Microneutralization Titers	None were significant				
Antibody Avidity	CD19+CD21+CD27+ (resting memory)	0.11 (0.44)	47	*0*.*29 (0*.*05)*	46
	CD19+CD21-CD27-CD20+ (tissue-like memory)	-0.23 (0.12)	47	**-0.32 (0.03)**	46
	CD19+CD21-CD27-CD20- (transitional)	-0.13 (0.40)	47	**-0.33 (0.03)**	46
B-cell memory (pH1N1 IgG ASC)	CD19+HLADR+CD38+ (activated)	-0.26 (0.16)	31	**-0.36 (0.04)**	33
	CD19+CD10+CD27+ (immature activated)	0.06 (0.71)	40	**0.40 (0.01)**	37
	*CD19+CD21+CD27+ (resting memory)*	*0*.*28 (0*.*08)*	40	**0.50 (0.002)**	37
	CD19+CD21+CD27-(naïve)	-0.09 (0.57)	40	*-0*.*28 (0*.*09)*	37
	CD19+CD21-CD27-CD20+ (tissue-like memory)	**-0.41 (0.01)**	40	-0.16 (0.35)	37
	CD19+CD21-CD27-CD20- (transitional)	-0.23 (0.16)	40	**-0.36 (0.03)**	37
	CD19+CD21-CD27+CD20- (plasmablasts)	*0*.*30 (0*.*06)*	40	0.05 (0.77)	37

High CD19+CD10+CD27+% (immature activated) and CD19+CD21+CD27+% (resting memory) were significantly correlated with increased IgG ASC post-dose 2 (ρ≥0.4, p≤0.01). Low CD19+HLADR+CD38+% (activated), and CD19+CD21-CD27-CD20-% (transitional) were also associated with high IgG ASC post-dose 2 (ρ = -0.36, p≤0.04), while low CD19+CD21-CD27-CD20+% (tissue-like memory) was associated with high IgG ASC post-dose 1 (ρ = -0.41, p = 0.01). There were marginal correlations between 1) high IgG ASC post-dose 1 and high CD19+CD21+CD27+% (resting memory) and CD19+CD21-CD27+CD20- (plasmablasts; ρ≥0.28, p≤0.08); and 2) low IgG ASC post-dose 2 and high CD19+CD21+CD27-% (naïve; ρ = -0.28, p = 0.09). In addition, at baseline, high IgG ASC was associated with high CD19+BAFFR+% (rho = 0.32; p = 0.046) (data not shown).

**Fig 4:**
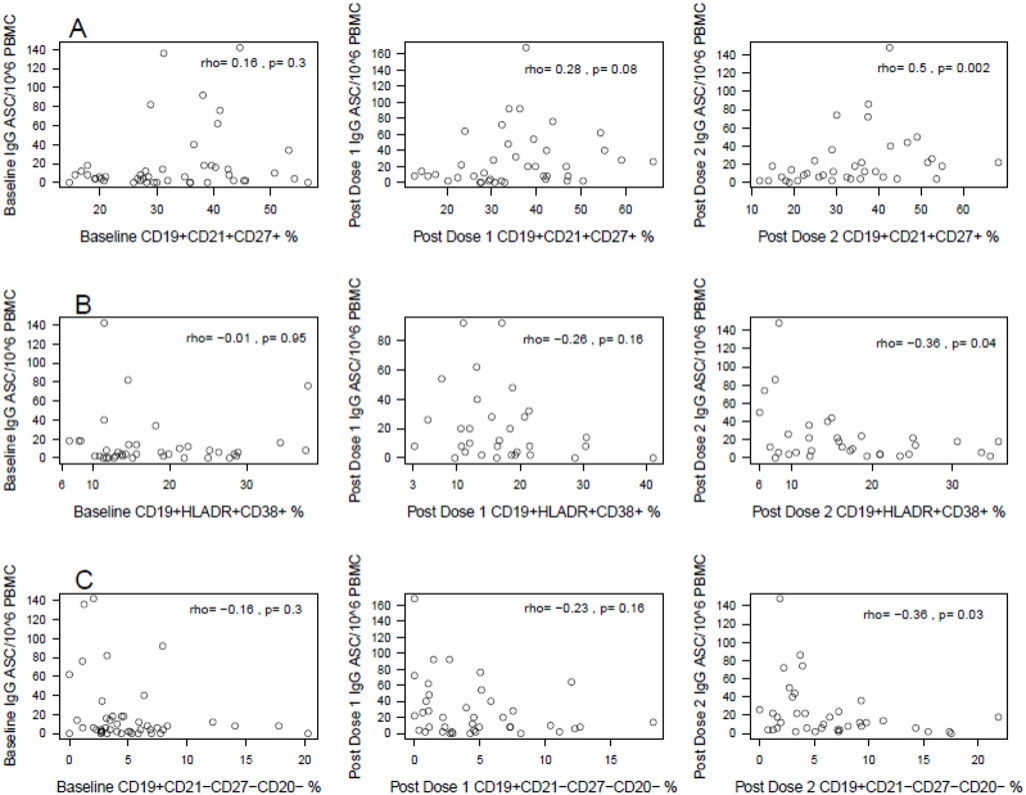
Correlations between pH1N1 Memory B Cells (IgG ASC) and Circulating B-Cell Subsets at Baseline and after Vaccination. Data are presented as scatter plots of pH1N1 IgG ASC/10^6^ PBMCs on the ordinate and select B-cell subsets on the abscissa. Each row shows correlations of a single B cell subset: CD19+CD21+CD27+ resting memory in the top row, CD19+HLADR+CD38+ activated in the middle, CD19+CD21-CD27-CD20- tissue-like on the bottom. Columns are organized by visit: baseline on the left, post-dose 1 in the middle and post-dose 2 on the right. The correlation coefficients and p values are shown on the graphs. The data show progression of the strengths of the correlations with each dose of vaccine.

It is interesting to note that for some phenotypes, such as CD19+CD21+CD27+% resting memory (Fig. 4A), CD19+HLADR+CD38+% activated (Fig. 4B) and CD19+CD21-CD27-CD20-% transitional B cells (Fig. 4C), the magnitude of the coefficient of correlation with IgG ASC progressively increased from baseline to post-dose 1 and 2 suggesting that the strength of the association between these B-cell subsets and perhaps their effect on the pH1N1-specific B-cell memory was best revealed by the administration of booster doses of the vaccine.

## Discussion

HIV-infected children and youth developed neutralizing antibodies, had an increase in AI and developed IgG B-cell memory after pH1N1 vaccination, demonstrating a full spectrum of B-cell responses to the vaccine. These findings are in agreement and complement our previous observations that HIV-infected children develop functional antibodies in response to seasonal influenza vaccine [37]. Taken together, these observations suggest that inactivated influenza vaccines can generally be expected to generate functional antibodies and B-cell memory in HIV-infected children and youth with relatively conserved or reconstituted CD4 cell numbers.

B-cell memory is a critical component of the protective mechanism of vaccines, whereby the vaccinee acquires the ability to mount a quick, anamnestic response when exposed to the pathogenic agent. A single administration of a double-dose of the pH1N1 vaccine was sufficient to generate maximal IgG B-cell memory responses in the HIV-infected participants in this study. The magnitude of the IgG and the lack of the IgA B-cell memory responses to pH1N1 were similar to those described in HIV-infected and uninfected adults in response to split-virion pH1N1 vaccine [38]. The magnitude of the IgG B-cell memory response in our substudy correlated with the HAI and neutralizing antibody titers and with the increase in AI after vaccination, suggesting that B-cell responses to the vaccine were coordinated in the HIV-infected subjects.

It is important to note that the functionality, measured by neutralization and avidity, and the memory of the antibody responses after pH1N1 immunization were higher in subjects with HAI titers ≥1:40 compared with those with titers <1:40 before vaccination. Older age also played a significant role in the neutralizing antibody response to vaccination, but only in the group with baseline titers <1:40. This suggests that presumed pre-existing exposure to pH1N1 or to seasonal H1N1 (sH1N1) with cross-reactive antibodies increased the immunogenicity of the vaccine. It is conceivable that previous exposure to influenza vaccines and/or infections conferred upon older children cross-reactive B- and/or T-cell memory that improved their responses to the pH1N1 vaccine. Although there was practically no B-cell epitope homology of the HA between the pH1N1 and the sH1N1 virus in the 2009 seasonal influenza vaccine (which circulated in 2007 and 2008 seasons) [39], there might be B-cell epitope homology of pH1N1 with sH1N1 circulating before 2007. Although homologies between pH1N1 and sH1N1 before 2007 have not been extensively investigated, this hypothesis is supported by a recent study that described age-associated cross-reactive antibody-dependent cellular cytotoxicity against pH1N1 and sH1N1 [40]. Another potential explanation is that the extensive T-cell epitope homology between pH1N1 and sH1N1 provided better T-cell help to the antibody responses to pH1N1 vaccine in individuals with higher sH1N1 exposure. This is in agreement with previous studies that found increased antibody responses to pH1N1 vaccines in individuals/animals with prior sH1N1 vaccination/infections [41–44]. We have previously shown a lack of association between the antibody response to pH1N1 vaccine and prior 2009 seasonal vaccine administration [32]. We also found a lack of association between pH1N1 and sH1N1 HAI titers at baseline. However, there was a significant association of pH1N1 and sH1N1 HAI titers after the 1^st^ and 2^nd^ pH1N1 immunizations (S4 Table). Other recent studies also described boosting of heterosubtypic antibodies by vaccination or infection [45–47]. These findings may be at least partially explained by cross-reactive T-cell stimulation of heterosubtypic memory B-cell antibody production.

Other findings of our study underscore the critical role of Th cells in the immunogenicity of the pH1N1 vaccine. pH1N1 HAI and neutralizing responses significantly correlated with pH1N1-specific IL-2 and/or IFNγ secretion after in vitro stimulation of PBMC. It is interesting to note that we did not find correlations of pH1N1-specific Th2-cytokine production, including IL-4, IL-5 or IL-13, with the magnitude or functionality of antibody responses or with IgG ASC. Other studies showed an association between IL-21 production and antibody responses to pH1N1 vaccine in HIV-infected individuals [13,14] that we could not reproduce. Furthermore, we could not detect an increase in pH1N1-specific IL-21 in culture supernatants after vaccination, although we detected significant increases in IL-2 and IL-4.

We did not find any correlations between the markers of vaccine response and CD4% or viral load in our analysis, likely due to the high rate of HAART utilization. This finding is important because these HIV characteristics represent the current standard of care for HIV-positive pediatric patients in the U.S. and in many places around the world.

We also sought to determine the relationship between the abnormal distribution of B-cell subsets that characterizes HIV infection and pH1N1 immunogenicity. We did not observe any changes in circulating B-cell subsets after vaccination. However, the frequency of high resting memory B cells were associated with high pH1N1 IgG ASC and AI after vaccination, whereas the frequency of tissue-like, transitional and/or HLADR+CD38+ activated B-cell subsets were associated with low pH1N1 IgG ASC and antibody avidity post-vaccination. Interestingly, high frequencies of activated immature B cells were associated with increased HAI and IgG ASC responses to the vaccine. There were no associations between the frequencies of TACI+, BAFF receptor+, or CXCR5+ B cells and responses to pH1N1 vaccine.

Our functional antibody studies showed age differences in the response to pH1N1 vaccines that were not detected by HAI in the parent study. Although the neutralizing antibody and HAI titers were highly correlated, only the neutralizing antibody response to the vaccine increased with age. The AI also had significantly higher gains over time in older compared with younger participants. Other studies of the functional antibody response post-pH1N1 vaccine or infection in the general population found significant differences between elderly adults (>60 or 65 years of age) and other age groups, but no differences between younger and older children [48,49]. There are multiple characteristics that set apart HIV-infected individuals from the general population. Characteristics that may explain the differential effect of age on the functional antibody responses to pH1N1 include: 1) higher attack rate of influenza infection in HIV-infected individuals [3] and formation of B- and T-cell memory to influenza antigens; 2) possibly higher rate of vaccination against influenza in HIV-infected children and youth due to regular visits to health care providers; and 3) higher dependence on recall (as opposed to primary) responses to influenza vaccines in HIV-infected compared with uninfected individuals [50].

We conclude that prior immunologic exposure to influenza vaccines and/or infections is likely to increase the responses to heterosubtypic influenza vaccines in HIV-infected children and youth. This effect may be mediated by cross-reactive memory B and/or Th cells. Taken together, the data suggest that repeat administration of influenza vaccine over multiple influenza seasons or within the same season may mitigate the poor immunogenicity of these products in HIV-infected children on HAART. Further studies with adjuvanted and live-attenuated vaccines are needed to identify optimal immunization regimens against influenza in this population.

## Supporting Information

S1 CONSORT Checklist CONSORT 2010 Checklist for current manuscript. (DOC)

S1 Protocol Original P1088 Protocol, parent study. (PDF)

S2 Protocol Amendment that was submitted and approved for the additional laboratory assays reported in this manuscript, also called New Works Concept Sheet 114 (NWCS 114). (DOCX)

S1 List List of all sites and their IRBs that participated in the original P1088 parent study. (DOCX)

S1 Table Characteristics of the NWCS 114 Sample compared to the Parent Study Population. (DOCX)

S2 Table Correlations of pH1N1 immune responses with HIV disease characteristics CD4% and HIV viral load. (DOCX)

S3 Table Phenotypic Characterization of B-Cell Subsets at Baseline and After pH1N1 Vaccination. (DOCX)

S4 Table Correlations between pH1N1 and sH1N1 HAI titers. (DOCX)
