# Supplementary material for: Characterization of Functional Antibody and Memory B-Cell Responses to pH1N1 Monovalent Vaccine in HIV-Infected Children and Youth
Source: PLoS One. 2015 Mar 18;10(3):e0118567. doi: 10.1371/journal.pone.0118567 (PMC4364897; doi:10.1371/journal.pone.0118567)
Supplement: S1 Protocol — (PDF) [file pone.0118567.s002.pdf]

IMPAACT P1088

A Phase II Study to Assess the Safety and Immunogenicity of an Inactivated **Influenza A (H1N1) 2009 Monovalent Vaccine** in HIV-1 Perinatally Infected Children and Youth

A Multi-Center, Domestic Trial of the
International Maternal Pediatric Adolescent AIDS
Clinical Trials Group (IMPAACT)

Sponsored by:
The National Institute of Allergy and Infectious Diseases (NIAID)
and
The Eunice Kennedy Shriver National Institute of Child Health and Human Development (NICHD)

Pharmaceutical Support Provided by Novartis,
Through BARDA (Biomedical Advanced Research and Development Authority),
Office of the Assistant Secretary for Preparedness and Response,
U.S. Department of Health and Human Services

BB-IND 14147

The IMPAACT Complications Scientific Committee Chair:	Sharon Nachman, M.D.
Protocol Chair:	Pat Flynn, M.D.
Protocol Co-Vice Chair:	Stephen Spector, M.D.<br>Coleen Cunningham, M.D.
NIAID Medical Officer:	Edward Handelsman, M.D.
DAIDS Program Officer:	Judi Miller, R.N., B.S.N.
NICHD Medical Officer:	George Siberry, M.D.
Clinical Trials Specialist:	Elizabeth Petzold, Ph.D.

Version 2.0
FINAL
April 23, 2010

IMPAACT P1088 TEAM ROSTER

All questions concerning this protocol should be sent via e-mail to actg.teamp1088@fstrf.org. Remember to include the subject's PID when applicable. The appropriate team member will respond to questions via e-mail with a "cc" to actg.teamp1088@fstrf.org. A response should generally be received within 24 hours (Monday - Friday). For protocol registration questions, e-mail protocol@tech-res.com or call 301-897-1707. Protocol registration material can be sent electronically to epr@tech-res.com or via fax at 1-800-418-3544 or 301-897-1701. For EAE questions, e-mail rccsafetyoffice@tech-res.com or call 1-800-537-9979 or 1-301-897-1709 or fax 1-800-275-7619 or 1-301-897-1710. To order study agent, call the Clinical Research Products Management Center at (301) 294-0741. For randomization or enrollment questions, contact the Data Management Center at 716-834-0900 or by email at sdac.random.desk@fstrf.org .

Protocol Chair

Pat Flynn, M.D.
St. Jude Children's Research Hospital
Department of Infectious Disease
262 Danny Thomas Place
MS 600
Memphis, TN 38105-2794
Phone: (901) 595-2338
Email: pat.flynn@stjude.org

Protocol Vice Chairs

Stephen Spector, M.D.
University of California, San Diego
Department of Pediatrics
Division of Infectious Diseases
Stein Clinical Research Building,
Room 430
9500 Gilman Drive, Mail Code 0672
La Jolla, CA 92093-0672
Phone: (858) 534-7055
E-mail: saspector@ucsd.edu

Coleen Cunningham, M.D.
Duke University Medical Center
DUMC Box 3499 Erwin Road
Room T915 Children's Health Center,
Duke Hospital
Durham, NC 27710
Phone: (919) 684-6335
Email: cunni016@mc.duke.edu

NIAID Medical Officer

Edward Handelsman, M.D.
HIV Research Branch
NIH, NIAID, DAIDS, TRP,
Room 5107
6700-B Rockledge Drive
Bethesda, MD 20892-7624
Phone: (301) 402-3221
E-mail: handelsmane@niaid.nih.gov

IMPAACT P1088 TEAM ROSTER

DAIDS Program Officer

Judi Miller, R.N., B.S.N.
NIH/NIAID
6700-B Rockledge Drive, MSC 7624
Room 5100
Bethesda, MD 20892-7624
Phone: (301) 496-1189
E-mail: jmillera@niaid.nih.gov

NICHD Medical Officer

George Siberry, M.D.
Pediatric Adolescent and Maternal AIDS (PAMA) Branch
Eunice Kennedy Shriver National Institute of Child Health and Human Development (NICHD)
National Institute of Health, DHHS
6700 Executive Blvd., Room 4B11H
MSC 7510
Bethesda, MD 20892-7510
Phone: (301) 496-7350
E-mail: siberryg@mail.nih.gov

Clinical Trials Specialist

Elizabeth Petzold, Ph.D.
IMPAACT Operations
Social & Scientific Systems
1009 Slater Road, Suite 120
Durham, NC 27703
Phone: (919) 287-4314
E-mail: epetzold@s-3.com

Wende Levy, R.N., M.S.
IMPAACT Operations
Social & Scientific Systems
8757 Georgia Avenue
Silver Spring, MD 20910
Phone: (301) 628-3384
E-mail: wlevy@s-3.com

Protocol Statisticians

Terence Fenton, Ed.D.
Principal Research Scientist
Statistical & Data Analysis Center
Harvard School of Public Health
Pediatric Section
651 Huntington Avenue
Boston, MA 02115-6017
Phone: (617) 432-3027
Email: fenton@sdac.harvard.edu

Petronella Muresan, M.S.
FSTRF-Harvard School of Public Health
900 Commonwealth Avenue, 2nd floor
Boston, MA 02215
Phone: (617) 632-2059
E-mail: petronm@sdac.harvard.edu

Protocol Data Manager

Barbara E. Heckman, B.S.
Frontier Science and Technology Research Foundation
4033 Maple Road
Buffalo, NY 14226-1056
Phone: (716) 834-0900, ext. 7231
E-mail: bheckman@fstrf.org

Westat Representative

Jennifer Bryant, M.P.A.
Westat
1441 W. Montgomery Ave., WB288
Rockville, MD 20850
Phone: (301) 294-3949
Email: jenniferbryant@westat.com

IMPAACT P1088 TEAM ROSTER

Protocol Immunologist
Adrianna Weinberg, M.D.
Departments of Pediatrics, Medicine and Pathology
Director, Clinical Virology Laboratory
University of Colorado Denver
Mail Stop 8604
12700 E. 19th Avenue, Room 11126
Aurora, CO 80045
Phone: (303) 724-4480
Email: adriana.weinberg@ucdenver.edu

DAIDS Pharmacist
Ruth Ebiasah, Pharm.D. M.S., R.Ph.
Pharmaceutical Affairs Branch
NIH, NIAID, DAIDS
6700-B Rockledge Drive, Rm 4223
Bethesda, MD 20892
Phone: (301) 402-0128
E-mail: ebiasahrp@niaid.nih.gov

Field Representative
Jill Utech, R.N., M.S.N., C.C.R.C.
St. Jude Children's Research Hospital
Department of Infectious Disease
262 Danny Thomas Place
MS 600
Memphis, TN 38105-2794
Phone: (901) 595-3490
Email: jill.utech@stjude.org

Investigator
Sharon Nachman, M.D.
SUNY at Stony Brook
Health Science Center
T11-080
Stony Brook, NY 11794
Phone: (631) 444-7692
Email: snachman@notes.cc.sunysb.edu

Laboratory Technologist

Patricia Anthony, CLS
Maternal, Child, and Adolescent
Virology Research Lab
University of Southern California
Keck School Of Medicine, Pediatrics
1801 E. Marengo Street
Los Angeles, CA 90033
Phone: (323) 226 4161
Email: patricia.anthony@usc.edu

Laboratory Data Coordinator

Anthony Bloom, M.S.
Frontier Science & Technology
Research Foundation
4033 Maple Road
Amherst, NY 14226
Phone: (716) 834-0900 x 7431
E-mail: bloom.anthony@fstrf.org

Community Advisory Board Representative

Dawn English, R.N.
Seattle Children's Hospital
16041 Wallingford Avenue North
Shoreline, WA 98133-5833
Phone: (206) 718-9479
Email: dawneme@msn.com

List of Commonly Used Abbreviations and Definitions

ONE (1) DOSE of vaccine	Each 30mcg vaccine dose consists of TWO separate 15mcg intramuscular (IM) injections
AAP	American Academy of Pediatrics
ACTN	AIDS Clinical Trials Network
AE	Adverse Event/Adverse Experience
ANC	Absolute Neutrophil Count
APIC	Association of Practitioners for Infection Control
ART	Antiretroviral therapy
ARV	Antiretroviral
BARDA	Biomedical Advanced Research & Development Authority, DHHS
CBC	Complete blood count
CDC	Centers for Disease Control and Prevention
CFR	Code of Federal Regulations
CIN	Cervical Intraepithelial Neoplasia
CMI	Cell-Mediated Immunity
CRF	Case Report Form
CRPMC	Clinical Research Products Management Center
CTL	Cytotoxic T Lymphocytes
DAIDS	Division of AIDS
DHHS	Department of Health and Human Services
DMC	Data Management Center
DTR	Deep Tendon Reflex
DMID	Division of Microbiology and Infectious Diseases
EAE	Expedited Adverse Event
eCRF	Electronic Case Report Form
Evaluable	Evaluable subjects are defined as having received two doses of **Influenza A (H1N1) 2009 Monovalent Vaccine** 21-28 days apart and had all HAI samples for collected up to and including Day 10 post Dose #2 within the protocol specified windows.
FDA	Food and Drug Administration
GBS	Guillain-Barré Syndrome
GCP	Good Clinical Practice
GMT	Geometric Mean Titer
H1N1	2009 Pandemic influenza A virus (swine flu)
HAART	Highly Active Antiretroviral Therapy
HA	Hemagglutinin
HAI	Hemagglutination Inhibition
HCG	Human Chorionic Gonadatropin

HBV	Hepatitis B Virus
ICH	International Conference on Harmonization
IEC	Independent or Institutional Ethics Committee
ILI	Influenza-like-illness
IMPAACT	International Maternal Pediatric Adolescent AIDS Clinical Trials Group
IND	Investigational New Drug Application
IRB	Institutional Review Board
ISM	Independent Safety Monitor
LAR	Legally Authorized Representative
MCG	Microgram
MedDRA ®	Medical Dictionary for Regulatory Activities
MN	Microneutralization Assay
N	Number (typically refers to subjects)
NCI	National Cancer Institute
NIAID	National Institute of Allergy and Infectious Diseases
NICHD	The Eunice Kennedy Shriver National Institute of Child Health and Human Development
NIH	National Institutes of Health
NICU	Neonatal Intensive Care Unit
NP	Naso-pharyngeal swabs
Nt	Neutralizing
OHRP	Office for Human Research Protections, DHHS
PI	Principal Investigator
QA	Quality Assurance
QC	Quality Control
RT-PCR	Reverse Transcriptase Polymerase Chain Reaction
SAE	Serious Adverse Event/Serious Adverse Experience
SDMC	Statistical and Data Management Center
SMC	Safety Monitoring Committee
TIV	Trivalent Influenza Vaccine
US	United States

SUMMARY OF CHANGES

All changes in this version appear in boldface type. Editorial changes including corrections of typographical errors and other changes required to update information that does not affect regulatory issues or patient consent may also be included. These changes contain all the information contained in Letter of Amendment #1, dated 10/13/09:

1. **The Influenza A (H1N1) 2009 Monovalent Vaccine manufactured by Novartis has been approved by the FDA. All references to the study product have been modified to indicate that the study vaccine is a licensed product and is no longer considered as investigational.**

2. **The vaccine is now referred to as "Influenza A (H1N1) 2009 Monovalent Vaccine" throughout the protocol. The virus is now referred to as "2009 H1N1 Influenza A virus" throughout the protocol. Similarly, all references to 'S-OIV' or 'swine-origin' have been removed.**

3. **The Table of Contents has been updated.**

4. **Schema and Section 2.2 – Secondary Objectives. An additional secondary objective has been added: "To explore factors related to HIV and its treatment that might affect the responses to H1N1 vaccinations."**

5. **Section 4.2 – Exclusion Criteria; Informed Consent: The Influenza A (H1N1) 2009 Monovalent Vaccine (Novartis) does not contain any of the following allergens: Gelatin, formaldehyde, octoxinol or chicken protein, but does contain neomycin and polymyxin. Therefore, exclusion criterion 4.21 has been updated to: "Has a known allergy to eggs, egg products, neomycin or polymyxin."**

6. **Section 4.23 – Exclusion Criteria: This is has been expanded to include a definition of H1N1 infection, and as such now reads: "Has a history of probable or proven pandemic 2009 H1N1 Influenza A infection prior to study entry, as defined as "Symptoms of influenza-like illness as well as a proven history, by RT-PCR, of novel influenza H1N1 infection, or, has positive influenza diagnostic testing since June 2009 (specificity to H1N1 not required) prior to study entry."**

7. **Section 4.7: Enrollment procedures have been updated.**

8. **Section 5.12; Needle Length Chart: It is recommended that all subjects in P1088 should have study vaccine administered in the deltoid muscle. Vaccinations with study vaccine that are to be administered in the lateral thigh should follow the instructions in the package insert.**

9. **Section 6.1 – Toxicity Management: The first bullet has been revised to read: "ALL grades of adverse events, whether or not associated with study vaccine, should be recorded on the appropriate CRF and will be assessed in the safety outcomes."**

10. **Section 6.2 and 6.5 – Subject Management: The following text has been added: "For this protocol, the following definition of H1N1 infection will be used: "Symptoms of influenza-like illness as well as a proven history, by RT-PCR, of novel influenza H1N1 infection, or, has positive influenza diagnostic testing since June 2009 (specificity to H1N1 not required) prior to study entry."**

    If a subject experiences documented H1N1 infection, as defined in Section 4.23, between Step I and Step II of the protocol, the subject should NOT receive the second dose of vaccine but should continue to be seen at the scheduled study visits, and have blood collected, as per the Schedule of Evaluations."

11. **Section 8.5 – Study Safety Monitoring: The following text has been added: "For safety monitoring, a vaccine related SAE is an SAE that is judged to be "definitely", "probably" or "possibly related" to study vaccine.**

12. **Appendix III – Informed Consent: The date of the Sample Informed Consent should read: Version 2.0 dated April 23 2010.**

13. **WHY IS THIS STUDY BEING DONE? (IC page 1/10)**
    Text has been modified to read: "H1N1 virus, historically known as 'swine flu virus', is a new strain of influenza (flu) virus that causes illness in people."

14. **WHY IS THIS STUDY BEING DONE? (IC page 1/10)**
    Text has been modified to read: "The purpose of this research study is to determine if this vaccine which has been licensed by the United States Food and Drug Administration (FDA) is safe and will help the body's normal defenses against the effects of H1N1 influenza in HIV-infected children and youth. The licensed vaccine to be used in this study may not prevent influenza virus infection, but may prevent severe disease and possibly death which has been seen with this H1N1 influenza virus in children."

15. **WHY IS THIS STUDY BEING DONE? (IC page 2/10)**
    Text has been modified to read: "This vaccine contains a trace amount of thimerosal (mercury)."

16. **WHY IS THIS STUDY BEING DONE? (IC page 2/10)**
    Text has been modified to read: "The vaccine is made with products from eggs."

17. **WHY IS THIS STUDY BEING DONE? (IC page 2/10)**
    Text has been modified to read: "If you are allergic to egg protein (eggs or egg products), you would not be able to receive this vaccine."

18. **WHY IS THIS STUDY BEING DONE? (IC page 2/10)**
    The following text has been removed:
    "Also, if you choose not to eat pork for religious reasons, you may want to consult your religious authority regarding getting a vaccine that contains products derived from pigs."

19. WHAT ARE THE RISKS OF THE STUDY? (IC Page 5/10)
The following text has been added:
"The dose of study drug is higher than the recommended dose for healthy children because children with HIV infection may not respond as well as healthy children to the lower dose of vaccine."

20. WHAT ARE THE RISKS OF THE STUDY? (IC Page 5/10)
Symptoms of vaccine related reactions should be modified to read:

- **skin rash (hives)**
- **fever**
- **nausea**
- **vomiting**
- **diarrhea**
- **abdominal pain**
- **cough**
- **sore throat**
- **achiness**
- **feeling tired (fatigue)**
- **general feeling of illness**
- **myalgia (muscle aches)**

Serious Vaccine Related Reactions:

- **Difficulty breathing**
- **Sweating**
- **Fast pulse**
- **Swelling around the mouth, throat or eyes**

21. After you have received the vaccine (IC Page 6/10)
"Myalgia (muscle aches)" has been added to the list of vaccine-related reactions.

22. WHAT OTHER CHOICES DO I HAVE BESIDES THIS STUDY? (IC page 8/10)
<u>Text has been modified to read:</u>
"Alternatives to your participation in this study include receiving from your health care provider the seasonal flu vaccine and Influenza A (H1N1) 2009 Monovalent Vaccine. Alternatively, you may choose not to receive any vaccine."

23. WHAT ARE THE RISKS OF THE STUDY? (IC page 5/10)
Text has been modified to read:
"Life-threatening allergic reactions to vaccines are very rare. If they do occur, it is usually within a few minutes to a few hours after the vaccination."

TABLE OF CONTENTS

SCHEMA 11

1.0 INTRODUCTION 13
1.1 Background 13
1.2 Rationale 16
1.3 Risks and Benefits of the Study 18

2.0 STUDY OBJECTIVES 18
2.1 Primary Objectives 18
2.2 Secondary Objectives 18

3.0 STUDY DESIGN 19

4.0 SELECTION AND ENROLLMENT OF SUBJECTS 20
4.1 Inclusion Criteria for Study Entry (STEP I) 20
4.2 Exclusion Criteria for Study Entry (STEP I) 21
4.3 Inclusion Criteria for STEP II 23
4.4 Exclusion Criteria for STEP II 23
4.5 Concomitant Medication Guidelines 24
4.6 Disallowed/Precautionary Medications 25
4.7 Enrollment Procedures 25
4.8 Co-enrollment Procedures 26

5.0 STUDY TREATMENT 26
5.1 Drug Regimens and Administration 26
5.2 Drug Formulation 28
5.3 Dose Modifications 29
5.4 Drug Supply, Distribution and Pharmacy 29

6.0 SUBJECT MANAGEMENT 29
6.1 Toxicity Management 29
6.2 Subject Management 30
6.3 Criteria for Deferral of Second Dose of Vaccine 31
6.4. Criteria for Permanent Exclusion from Second Dose of Vaccine 32
6.5 Evaluation of Influenza-Like Illness (ILI) 32
6.6 Evaluation of Possible Guillain-Barré Syndrome 32
6.7 Management of Pregnancy on Study 32
6.8 Permanent Study Discontinuation 33

7.0 EXPEDITED ADVERSE EVENT REPORTING 34
7.1 Expedited Adverse Event Reporting to DAIDS 34

8.0 STATISTICAL CONSIDERATIONS 35
8.1 General Design Issues 35
8.2 Endpoints and Outcome Measures 36
8.3 Randomization and Stratification 37
8.4 Sample Size and Accrual 37
8.5 Safety Monitoring 37
8.6 Analyses 39

9.0 HUMAN SUBJECTS 42
9.1 Institutional Review Board and Informed Consent 42
9.2 Subject Confidentiality 43
9.3 Risks and Benefits of the Study 43
9.4 Study Discontinuation 45

10.0 PUBLICATION OF RESEARCH FINDINGS 45

11.0 BIOHAZARD CONTAINMENT 45

12.0 REFERENCES 46

APPENDICES:

IA SCHEDULE OF EVALUATIONS

IB STEP II-SCHEDULE OF EVALUATIONS FOR WOMEN WHO BECOME PREGNANT BETWEEN DOSE #1 and DOSE #2 of STUDY VACCINE AND THEIR INFANTS

II PUBLIC READINESS AND EMERGENCY PREPAREDNESS ACT (PREP Act)

III DAIDS SAMPLE INFORMED CONSENT

SCHEMA

A Phase II Study to Assess the Safety and Immunogenicity of an Inactivated **Influenza A (H1N1) 2009 Monovalent Vaccine** in HIV-1 Perinatally Infected Children and Youth

DESIGN: Multi-center, Phase II, open label study
STEP I: Dose #1 of study vaccine
STEP II: Dose #2 of study vaccine

NOTE: A dose is defined as TWO separate 15mcg intramuscular (IM) vaccine injections, totaling 30mcg

SAMPLE SIZE: 140 children and youth to obtain at least 120 evaluable* subjects

*Evaluable subjects are defined as having received two doses of **Influenza A (H1N1) 2009 Monovalent Vaccine** 21-28 days apart and had all HAI samples collected up to and including Day 10 post Dose #2 within the protocol specified windows.

POPULATION: HIV-1 perinatally infected** children and youth (≥ 4 years to <25 years of age). Subjects not on ARV therapy should not have received ARVs within 90 days prior to vaccination nor plan to initiate ARVs within the next 60 days. Subjects on ARVs must have been on a stable ARV regimen for at least 90 days prior to vaccination with no intention of modifying the regimen within the next 60 days.

**For inclusion into the study, if perinatal acquisition of HIV infection cannot be confirmed based on the child's medical record, it is acceptable to enroll the child if the investigator's assessment is that the most likely route of infection was perinatal.

STRATIFICATION: Subjects will be stratified by age (at least 40 evaluable subjects per group)
Group 1: ≥ 4 years to < 9 years
Group 2: ≥ 9 years to <18 years
Group 3: ≥ 18 years to < 25 years

REGIMEN: In Step I, the initial dose of **Influenza A (H1N1) 2009 Monovalent Vaccine** will be administered to all eligible subjects during the study entry visit (Day 0) as a 30mcg dose; (given as TWO 15mcg intramuscular injections). Step II is the second dose of **Influenza A (H1N1) 2009 Monovalent Vaccine** as a 30mcg dose, administered as TWO 15mcg IM injections, 21-28 days after vaccine Dose #1. If a woman becomes

pregnant prior to Dose #2, she will not receive Dose #2 at Day 21, but will defer vaccine Dose #2 to ≥ 14 weeks of gestation.

Note: A second set of inclusion/exclusion criteria must be met prior to administration of Dose #2.

STUDY DURATION: All subjects will be followed for 7 months from enrollment. Women, who become pregnant before receiving Dose #2 of study vaccine, will have study vaccine held until they are ≥ 14 weeks of gestation, and then they will be followed until up to 28 days after delivery. Their infant(s) will have a study visit within the first 28 days of life.

OBJECTIVES:

Primary

1. To determine the safety and immunogenicity after each of two doses of **Influenza A (H1N1) 2009 Monovalent Vaccine** in HIV-1 perinatally infected children and youth.

Secondary

1. To correlate vaccine response with baseline antibody titer.

2. To assess persistence of antibody response 6 months after Dose #2 of **Influenza A (H1N1) 2009 Monovalent Vaccine**.

3. To correlate immune responses with CD4+ cell count, CD4%**,** ARV use, plasma HIV-1 RNA concentration at the time of first immunization, and with timing of seasonal influenza vaccine.

4. To assess cell-mediated immune responses to **Influenza A (H1N1) 2009 Monovalent Vaccine**.

5. To describe influenza-like illness (ILI) occurring during the course of the study period, through clinic visits for all acute febrile and respiratory illnesses, including collection of respiratory specimens for determination of influenza with RT-PCR.

6. **To explore factors related to HIV and its treatment that might affect the responses to H1N1 vaccinations."**

1.0 INTRODUCTION

1.1 Background

The Nature of Influenza Pandemics.

The dissemination of animal influenza A viruses in human populations [including subtypes A/H5N1, H7N7, H9N2 and, most recently, H1N1 swine-origin influenza virus; also designated "**2009 H1N1 Influenza A**" herein] has added urgency to ongoing efforts to develop plans for responding to potential influenza pandemics (1-4). Three pandemics have occurred during the last century. During the 1918 influenza A/H1N1 pandemic, an estimated 40 million deaths occurred worldwide (5;6). Excess mortality, high morbidity, and social disruption were all noted during the 1957 influenza A/H2N2 and the 1968 influenza A/H3N2 pandemics (7). In both of these pandemics, human populations lacked significant levels of pre-existing immunity to the pandemic virus, resulting in rapid spread of the novel influenza A virus subtypes. Thus, the emergence of a new influenza subtype in a human population has the potential to produce a public health emergency. The **2009 H1N1 Influenza A** has reached pandemic levels as defined by the World Health Organization ("an influenza virus that is causing sustained community level outbreaks in at least two countries in at least two WHO regions") (8). Animal models indicate that **2009 H1N1 Influenza A** virus replicates better in the lung and the gastro-intestinal tract compared with seasonal H1N1, suggesting that the pandemic virus may cause higher morbidity than the seasonal one. The virulence of the pandemic strain in humans is still uncertain.

The cornerstones of pandemic influenza preparedness include enhanced surveillance for the identification of emerging viruses, expanded capacity to produce and deliver relevant vaccines, availability of antiviral medications for prevention and treatment of infections caused by pandemic viruses, and improved public health infrastructure to manage and coordinate control efforts. The threat of pandemic influenza in 1976 (swine influenza) and again in 1977 (Russian influenza) resulted in an unadjuvanted inactivated influenza virus vaccine development program that provided important insights into variables influencing the immune responses to immunization (5;6). These included dosage of hemagglutinin (HA) in the vaccine, the number of doses administered to a vaccinee (1 or 2), and the type of vaccine administered (whole virus, split virus, or purified surface antigen). Host factors that influence immune responses included age, prior priming, and presence of underlying disease and immunosuppressive medications.

Immune Response to Influenza Vaccines.

Hemagglutinin (HA) is the viral receptor that binds to the host's epithelial cell receptor, allowing the virus to enter the host cell. Hence, antibodies to the HA play a major role in protective immunity to influenza virus infection (9;10), and are the basis for the licensure of influenza vaccines Resistance to infection with seasonal influenza virus strains correlates directly with both serum hemagglutination inhibition (HAI) and neutralizing

(Nt) antibody levels. Measurements of serum HAI and Nt antibodies are used to assess the immunogenicity of both seasonal and pandemic influenza vaccines (9;10).

The magnitude of the immune response to a unadjuvanted inactivated influenza vaccine is greatly affected by the dosage of antigen (usually expressed as weight of HA) contained in the vaccine. Studies evaluating the effect of HA dosage on immune responses to seasonal unadjuvanted inactivated influenza vaccines performed over the past 35 years demonstrated dose-related increases in serum and mucosal antibody responses (10-18). Higher vaccine dosages are also associated with the development of higher levels of serum antibodies that recognize antigenically distinct drift variants (18) and can overcome suboptimal responses in immunologically challenged vaccinees, such as elderly patients (17;19;20).

The effect of HA dosage on the immunogenicity of inactivated vaccines targeting some potential pandemic influenza virus strains (H9N2, H5N1) has been evaluated (21-23). Although higher doses of HA routinely induce a significantly greater response than a single dose, the response may still be poor if the antigen is not highly immunogenic. For example, H5N1 vaccines are much less immunogenic than seasonal vaccines or H9N2 vaccines, and two doses of 90mcg of either recombinant baculovirus-expressed H5N1 HA or of an inactivated subvirion antigen vaccine elicited antibody responses in only approximately 50% of young healthy adult subjects (21;22). Higher dosages of HA are also associated with more frequent adverse events.

The route of administration may affect the immune response. Intranasal administration of live attenuated or virosomal vaccines has been purported to enhance local responses including those of HIV-1 infected patients (24;25). Intradermal administration of unadjuvanted inactivated influenza vaccines generates higher antibody titers compared with intramuscular administration of the same HA-containing preparations (26-28).

The use of adjuvants is another approach to improve the immunogenicity of influenza vaccines (29;30). Adjuvants have the potential to improve serum immune responses at a given dose of antigen, to decrease the amount of antigen needed in the vaccine (dose-sparing), and to improve the immune responses by groups that generally respond poorly to inactivated antigens (e.g., immunocompromised, elderly) (31). New and more potent adjuvants have not been evaluated in HIV-infected patients.

<u>Novel Pandemic Strain of Influenza.</u>
Recently, a novel swine-origin influenza A/H1N1 virus, now designated **2009 H1N1 Influenza A**, was identified as a significant cause of febrile respiratory illnesses in Mexico and the United States (US) (3;4;6). In a recent report of severe respiratory disease concurrent with the circulation of **2009 H1N1 Influenza A** in Mexico, individuals of all ages were affected. However, compared to prior influenza seasons, the greatest increase in mortality occurred in children and young adults. It rapidly spread to many countries around the world, prompting the World Health Organization to declare a pandemic on June 11, 2009 (8). Serologic data from several cohorts in different age groups that

received the licensed seasonal trivalent influenza vaccine (TIV) suggest that these vaccines are unlikely to provide protection against the new virus (32). However, approximately 33% of adults older than 60 years of age have measurable levels of serum HAI or Nt antibody against the **2009 H1N1 Influenza A**, whereas young adults and children completely lack protective titers. These data indicate the need to develop vaccines against the new **2009 H1N1 Influenza A** strain that will be suitably protective for persons in different age groups; especially for those with one or more risk factors for severe disease.

<u>Influenza in HIV-1 Infected Patients.</u>
HIV-1 infected patients not only suffer the morbidity typical of seasonal influenza and potential interference with their HIV therapy, but there is also the potential for influenza infection that is more severe than that typical of age-matched uninfected people (33;34). Madhi et al have reported that severe lower respiratory tract infections are significantly more likely to occur in HIV-infected children than in HIV-uninfected children (RR 8.03, 95% CI: 5.05-12.76) (35). HIV-1 infected patients may also shed virus for greater periods of time and demonstrate continued infectivity and necessitate isolation in the clinic or hospital (36).

<u>Response to Seasonal Influenza Vaccine in HIV-1 Infected Patients.</u>
The antibody responses to seasonal TIV are blunted in HIV-1 infected children and adults who are not receiving antiretroviral therapy (ART) (37-39). In patients who do not have progressive HIV-1 disease and/or are receiving highly active ART (HAART), these responses are improved (40-42). Efficacy of seasonal influenza vaccine in HIV-infected patients has been established in four controlled trials (43).

<u>Guillain-Barré Syndrome Associated with Influenza Vaccine.</u>
Guillain-Barré syndrome has not typically been considered a complication of seasonal influenza vaccination. However, an excess of Guillain-Barré syndrome was reported in association with vaccine against the novel 1976 swine influenza strain that prompted mass immunization in preparation for a possible pandemic (44;45). National surveillance uncovered a total of 1098 patients with onset of Guillain-Barré syndrome from October 1, 1976, to January 31, 1977. A total of 532 patients had recently received an A/New Jersey influenza vaccination prior to their onset of Guillain-Barré syndrome. The estimated attributable risk of vaccine-related Guillain-Barré syndrome in the adult population was just under one case per 100,000 vaccinations (46). This experience will dictate certain features of the safety assessment planned for this protocol.

This protocol has been developed in collaboration with the Department of Microbiology and Infectious Diseases (DMID). DMID has sponsored a parallel study of **Influenza A (H1N1) 2009 monovalent vaccine** in healthy children in the United States. Vaccine timing, immunogenicity assays and vaccine safety measures will occur in both studies. Significant toxicity events in one study will impact all ongoing H1N1 vaccine studies.

1.2 Rationale

1.21 Overview

1. HIV infection increases the risk of complications from influenza infection, especially secondary bacterial infections.
2. Data with other vaccines such as Hepatitis B virus (HBV) vaccine have suggested that increasing antigen titer of the vaccine dose improves antibody response in HIV infected individuals. Higher doses of inactivated HBV vaccine have been shown to improve antibody response in HIV-infected youth (47).
3. Data from other populations suggests an increased antibody response to higher dosed seasonal influenza vaccine.
4. The P1088 study team believes that HIV-1 infected children and youth are at increased risk of severe illness and death from the swine-origin H1N1 influenza virus, since their HIV infection increase their risk of poor response to dosages of vaccine that are immunogenic in other populations. Use of a higher dose (30mcg) of **Influenza A (H1N1) 2009 monovalent vaccine** in this study of HIV-1 infected children and youth, will achieve scientific objectives of timely assessment of safety and immunogenicity in these high-risk populations while maximizing the potential for study participants to be protected by study vaccine from **2009 H1N1 Influenza A** virus this season.

1.22 Justification Discussion

It is very likely that the continuing epidemic spread of **2009 H1N1 Influenza A** infection in the US will greatly increase in the fall-winter season when social interactions and climactic conditions become even more conducive to spread of influenza viruses. While reports to date suggest that healthy individuals typically have a mild illness, underlying medical conditions including immunodeficiency appear to increase risk for severe disease and even death with pandemic H1N1 (48). Thus, knowledge of the safety and immunogenicity of the **Influenza A (H1N1) 2009 monovalent vaccine** in HIV-infected children and youth is critically important to address the health care needs of this vulnerable population.

Because seasonal influenza has been shown to cause more severe illness in HIV-infected individuals compared to that typical of age-matched uninfected people (33;34), it is likely that **2009 H1N1 Influenza A** will result in significant morbidity and possibly mortality in HIV infected individuals. Morbidity may be a direct result of the influenza virus or infection may result in secondary bacterial infections or decreased adherence to the patient's antiretroviral therapy due to the severe nausea and vomiting that may occur with pandemic H1N1 illness. Prevention of infection in this population will be critical.

It is well established that seasonal influenza infection impacts children in a community before becoming widespread in adult populations. Susceptibility to disease among young populations appears even more pronounced with **2009 H1N1 Influenza A** as one third of older adults have measurable levels of serum HAI or neutralizing antibody against the **2009 H1N1 Influenza A** while young adults and children completely lack protective titers. The serologic data is consistent with the observation that the attack rate and disease severity for the virus appears to be much higher in younger populations with relative protection of those >50 years of age.

Efforts are currently underway to evaluate **Influenza A (H1N1) 2009 monovalent vaccine** in healthy children. However, HIV-1 infected children attend schools and participate in all the activities that typically put children at such high risk for infection with influenza. Protection of HIV-1 infected children and youth from **2009 H1N1 Influenza A** will require knowledge of safety and immunogenicity of these new products in this population.

This study will assess the safety and immune response following each of the two doses of Novartis **Influenza A (H1N1) 2009 monovalent vaccine** in HIV-1 infected children and youth in the US and Puerto Rico. Two doses are thought to be required because study subjects have had no prior exposure to **2009 H1N1 Influenza A**. Because seasonal influenza vaccine often results in blunted response in HIV-1 infected persons, we have opted to investigate the higher dose of antigen, 30mcg, in comparison to the 15mcg dose that is currently being studied in ongoing trials of the **Influenza A (H1N1) 2009 monovalent vaccine** in healthy children. We have also opted to stratify our study population into 3 groups based on age. The groups were selected to provide information across all age groups and with knowledge that there would be insufficient power to compare immune response across age groups. This study is limited to HIV-1 perinatally infected children and youth.

In order to understand the mechanism of disease and protection, we will investigate the seroresponse, duration of response, and development of influenza-like illness following vaccine in this population. We also propose investigation of the cell-mediated response to vaccine. The generation of cytotoxic T lymphocyte (CTL) responses against **2009 H1N1 Influenza A** is of particular interest, because this virus replicates better in lung tissue than seasonal influenza and CTLs are the major mediator of viral clearance in the lungs. Memory B cells to **2009 H1N1 Influenza A** will ensure that the host responds adequately to exposure to the wild type virus.

In summary, **2009 H1N1 Influenza A** is likely to infect a significant proportion of HIV-1 infected children and youth if an effective vaccine is not available

before infection is widespread. Infection will likely lead to severe disease in this vulnerable population, therefore, vaccine efforts are critical. Immunogenicity of the candidate **Influenza A (H1N1) 2009 monovalent vaccine** must be established in HIV-1 infected children in order to assure that this population is protected. Lack of a protective immune response would support the need for additional measures to protect this high risk population.

1.3 Risks and Benefits of the Study

See section 9.3 for detailed information on risks and benefits of the vaccine.

2.0 STUDY OBJECTIVES

2.1 Primary Objectives

1. To determine the safety and immunogenicity after each of two doses of **Influenza A (H1N1) 2009 monovalent vaccine** in HIV-1 perinatally infected children and youth.

2.2 Secondary Objectives

1. To correlate vaccine response with baseline antibody titer.

2. To assess persistence of antibody response 6 months after Dose #2 of **Influenza A (H1N1) 2009 monovalent vaccine**.

3. To correlate immune responses with CD4+ cell count, CD4%**,** ARV use, plasma HIV-1 RNA concentration at the time of first immunization, and with timing of seasonal influenza vaccine.

4. To assess cell-mediated immune responses to **Influenza A (H1N1) 2009 monovalent vaccine**.

5. To describe influenza-like illness (ILI) occurring during the course of the study period, through clinic visits for all acute febrile and respiratory illnesses, including collection of respiratory specimens for determination of influenza with RT-PCR.

6. **To explore factors related to HIV and its treatment that might affect the responses to H1N1 vaccinations.**

3.0 STUDY DESIGN

This is a multi-center, open label, Phase II study in 140 children and youth, aged ≥ 4 years to <25 years old in the US and Puerto Rico, who were infected with HIV-1 via perinatal transmission. The purpose of the study is to investigate the safety, reactogenicity, and immunogenicity of a two dose regimen of **Influenza A (H1N1) 2009 monovalent vaccine**.

Subjects do not need to be receiving ARV therapy but if they are receiving ARVs, they must have been on a stable ARV regimen for at least 90 days prior to vaccination with no intention to modify the regimen within the next 60 days. Changes in dosage due to weight changes or formulation are acceptable. The site should contact the team (actg.teamp1088@fstrf.org) for other modifications. Additionally, if they are not currently receiving ARVs, the subject should not have received ARVs within 90 days prior to vaccination nor plan to initiate ARVs within the next 60 days.

STEP I: Subjects who meet the entry criteria for the study will receive **Influenza A (H1N1) 2009 monovalent vaccine**, 30 mcg, administered as TWO 15mcg IM injections, in either the deltoid muscle(s) or antero-lateral thigh muscle(s).* Dose #1 will be administered on Day 0.

STEP II: Eligible subjects will receive a second dose of **Influenza A (H1N1) 2009 monovalent vaccine**, 30 mcg, administered as TWO 15mcg IM injections, in either the deltoid muscle(s) or antero-lateral thigh muscle(s).* Dose #2 will be administered on Day 21 (+ 7 day window).

*Only children and teens (4-18 years) may receive an injection in the antero-lateral thigh muscle (refer to the table in section 5.12 “Injection Site and Needle Size”).

NOTE: For women who are found to be pregnant after receiving study vaccine Dose #1 but before study vaccine Dose #2, STEP II will be deferred until the woman is ≥ 14 weeks gestation. These women and their infant(s) will be followed as described in Appendix IB. Women who become pregnant after the second dose of vaccine will continue to be followed as outlined in Appendix IA.

Results of routine laboratory tests will be shared with the subject when they become available. The results of special laboratory tests, including HAI serology, other immunogenicity studies, and assessment of respiratory samples during influenza-like illness (ILI), will be batch tested, and not available until after the completion of the study. Any blood samples that are not used after 3 years following the end of the study analysis will be destroyed.
Immunogenicity of the vaccine will be primarily assessed by specific HAI titers in serum collected at baseline, after each of the 2 doses of the vaccines and at 7 months after enrollment. The assay was adapted from previously described assays developed and validated for the seasonal influenza viruses. B-cell and T-cell ELISPOT and CTL against **2009 H1N1 Influenza A virus** will be performed in a subgroup of subjects using peripheral blood mononuclear cells

cryo-preserved at the sites from blood collected at the same time points as the HAIs. These assays are also adapted from assays previously developed for seasonal influenza. At baseline and at 7 months, HAI titers and T-cell ELISPOT for the viruses contained in the seasonal vaccine and other antigens will also be measured.

The schedules of laboratory and clinical evaluations for this study are outlined in Appendices IA and IB, "Schedule of Evaluations".

NOTE: Other H1N1 influenza vaccines are currently under study in HIV-uninfected adults, children and youth. These vaccines include both 15mcg and 30mcg doses of adjuvanted and unadjuvanted, inactivated **Influenza A (H1N1) 2009 monovalent vaccines** from Novartis, as well as vaccines from other manufacturers. If one of the other ongoing NIH trials identifies a different preparation to be sufficiently safe and immunogenic, an additional cohort of 40 HIV-1 positive children and youth may be added to the P1088 study. However, this study will complete enrollment and vaccination of the subjects at the 30 mcg dose of Novartis study vaccine. If a new cohort is added, subjects will be followed as per the Schedule of Evaluations.

4.0 SELECTION AND ENROLLMENT OF SUBJECTS

4.1 Inclusion Criteria for Study Entry (STEP I)

4.11 Children and youth ≥ 4 years to <25 years of age at study entry.

4.12 HIV infection, defined as positive test results obtained from 2 different samples. Tests may include two of the same type OR two different types of tests listed below, as long as there are positive test results obtained from 2 different samples:

- HIV-1 antibody (ELISA + WB), obtained at age >18 months
- HIV-1 culture, any age
- HIV-1 DNA PCR, any age
- HIV-1 RNA PCR >10,000 copies/mL, any age
- Neutralizable HIV-1 p24 antigen obtained >28 days of age

4.13 In the opinion of the investigator, the route of HIV-1 transmission is perinatally acquired.

4.14 Subjects who are receiving ARVs must have been receiving a stable regimen for 90 days prior to entry with no intention to modify their regimen within 60 days following study entry.

4.15 Subjects who are not receiving ARVs at entry must not have received ARVs within 90 days prior to entry and must NOT plan to initiate ARVs within 60 days following study entry.

4.16 Ability to complete all study immunizations and evaluations, in the opinion of the investigator.

4.17 Females of child-bearing potential must have a negative pregnancy test within 72 hours prior to enrollment.

NOTE: "Female of child-bearing potential" is defined as girls who have reached menarche and have not undergone surgical sterilization (e.g., hysterectomy, or bilateral oophorectomy, or bilateral salpingotomy or tubal ligation). Acceptable documentation of lack of reproductive potential for females in this study is: lack of onset of puberty (by participant or care giver reported history or by physical examination), lack of menses (by participant or care giver reported history), or woman's self-reported history of surgical sterilization.

4.18 Subjects (and/or their partners) who are participating in sexual activity that could lead to pregnancy must agree to use at least one of the following methods of contraception as long as they are in this study:

- hormonal birth control drugs (oral, injectable or transdermal)
- male or female condoms with or without a spermicide
- diaphragm/cervical cap with spermicide
- intrauterine device (IUD).

Condoms are recommended because their appropriate use is the only contraception method effective for preventing HIV-1 transmission.

4.19 Subjects should have a documented platelet count of >50,000 per mm^3 and an ANC of >500 per mm^3 within the 30 days prior to study entry.

4.110 Youth of legal age (≥18 to < 25 years of age), parent or legal guardian, or subjects who are emancipated minors, who are willing and able to provide signed informed consent.

4.2 Exclusion Criteria for Study Entry (STEP I)

4.21 Has a known allergy to eggs**, egg products, neomycin or polymyxin.**

4.22 Has a history, in the opinion of the site investigator, of severe reactions following previous immunization with seasonal influenza vaccines that would contraindicate receipt of any influenza vaccine.

4.23 Has a history of probable or proven pandemic **2009 H1N1 Influenza A** infection prior to study entry**, as defined as "Symptoms of influenza-like illness as well as a proven history, by RT-PCR, of novel influenza H1N1 infection, or, has**

positive influenza diagnostic testing since June 2009 (specificity to H1N1 not required) prior to study entry."

4.24 Has received any live licensed vaccine within 4 weeks, or inactivated licensed vaccine within 2 weeks, prior to study entry.

4.25 Has received a non-licensed agent (vaccine, drug, biologic, device, blood product, or medication) within 4 weeks prior to study entry, or expects to receive another non-licensed agent during the course of the study.

4.26 Has an acute illness and/or a documented temperature greater than or equal to 100.0°F within 24 hours prior to study entry.

4.27 Use of anti-cancer chemotherapy or radiation therapy within the 36 months preceding study entry, or has immunosuppression as a result of an underlying illness or treatment (other than HIV-1 infection).

4.28 Has an active neoplastic disease.

4.29 Long term use of glucocorticoids, including oral or parenteral prednisone or equivalent (≥ 2 mg/kg per day or ≥ 20 mg total dose) for more than 2 weeks in the past 6 months, or high-dose inhaled steroids (>800 mcg/day of beclomethasone dipropionate or equivalent) within the preceding 6 months (nasal and topical steroids are allowed).

4.210 Has received immunoglobulin or other blood products within the 3 months prior to study entry.

4.211 History of Guillain-Barré Syndrome in the subject or subject's family (parents, siblings, half siblings, or children).

4.212 Onset of a neurological disorder including (but not limited to) absent ankle and patellar deep tendon reflexes in both legs (all four absent) within the past 6 months.

4.213 Disproportionate loss of strength in lower extremity or extremities, compared to the upper extremities within the past 6 months.

4.214 Has any condition that would, in the opinion of the site investigator, place the subject at an unacceptable risk of injury or render the subject unable to meet the requirements of the protocol.

4.3 Inclusion Criteria for STEP II

4.31 Received the first dose of **Influenza A (H1N1) 2009 monovalent study vaccine** at least 21 days ago.

4.32 Has a documented platelet count of >50,000 per mm^3 and an ANC of >500 per mm^3 within the 30 days prior to Step II entry.

4.33 Females of child-bearing potential must have a negative pregnancy test within 72 hours of Step II entry.

OR

If a woman became pregnant after Dose #1, she must be ≥14 weeks of gestation and have her obstetrician's permission to receive the vaccine.

NOTE: For management of women, who become pregnant after Dose #1, see section 6.7.

4.4 Exclusion Criteria for STEP II

If a subject meets any of the following exclusion criteria, they should not receive Dose #2 (see section 6.4).

4.41 Has received a non-licensed agent (vaccine, drug, biologic, device, blood product, or medication), other than from participation in this study, since Dose #1, or expects to receive another non-licensed agent before the end of the study.

4.42 Use of anti-cancer chemotherapy or radiation therapy since Dose #1, new diagnosis of an active malignancy, or is immunosuppressed as a result of an underlying illness (other than HIV-1 infection) or treatment.

4.43 Use of glucocorticoids, including oral or parenteral steroids (≥ 2 mg/kg per day or ≥ 20 mg total dose) for more than 2 weeks since vaccine Dose #1, or high-dose inhaled steroids (>800 mcg/day of beclomethasone dipropionate or equivalent) since Dose #1 (nasal and topical steroids are allowed).

4.44 Has received immunoglobulin or other blood products since Dose #1.

4.45 Any Grade 3 toxicity or adverse event experienced by a subject unless the investigator has received protocol team approval.

4.46 Any Grade 4 toxicity or adverse event (other than injection site reaction or fever) that is definitely, probably or possibly related to study vaccine.

4.47 Any Grade 4 injection site reactions or fever experienced by a subject, unless the investigator has received protocol team approval.

4.48 Any Grade 4 adverse events that are definitely not or probably not related to study vaccine, unless the investigator has received protocol team approval.

4.49 New occurrence or new awareness of Guillain-Barré Syndrome in the subject or subject's family (parents, siblings, half siblings, or children) since Dose #1.

4.410 A new onset of a neurological disorder including (but not limited to) absent ankle and patellar deep tendon reflexes in both legs (all four absent) since Dose #1.

4.411 Disproportionate loss of strength in lower extremity or extremities, compared to the upper extremities (not thought to be related to pregnancy) since Dose #1.

4.412 Documented **2009 H1N1 Influenza A infection** since Dose #1 **(see 4.23 for definition)**

4.413 The subject, parent, or guardian refuses further vaccination.

4.414 Any new disease which the investigator judges to be clinically significant or clinically significant findings since Dose #1 that, in the investigator's opinion, would compromise the safety of the subject.

4.415 Subject withdraws consent**.** The subject may withdraw their consent for study participation at any time and for any reason, without penalty.

4.5 Concomitant Medication Guidelines

Administration of any medication, therapies (including anti-viral treatments for influenza or influenza prophylaxis), or vaccines will be documented in the study case report forms (CRFs). All ARVs and vaccine products administered during the 28 days prior to study entry through the end of study, or early termination, will be collected. All other medications will be collected from 28 days prior to study entry to 10 days after Dose #2.

4.51 Vaccines

Receipt of any vaccines besides the study product will be collected throughout the study from enrollment to the off study visit. The administration of inactivated licensed vaccines, including inactivated seasonal influenza TIV, should be at least

2 weeks (14 days) before Dose #1 or delayed until the STEP II Day 10 visit has occurred and immunogenicity samples have been collected.

The administration of live licensed vaccines, including seasonal cold-adapted live influenza vaccine, should be at least 4 weeks (28 days) before Dose #1 or delayed until at least the STEP II Day 10 visit has occurred and immunogenicity samples have been collected.

If administration of the seasonal influenza vaccine outside of the windows described above is necessary for clinical care, the investigator should notify the protocol team at actg.teamp1088@fstrf.org. The seasonal influenza vaccine is not provided by this study.

4.6 Disallowed/Precautionary Medications

The following medications should be avoided, if possible, and alternative treatments sought. Medications that might interfere with the evaluation of the investigational product should not be used unless absolutely necessary. Medications in this category include, but are not limited to:

- Glucocorticoids, i.e., oral, parenteral and high-dose inhaled steroids,
- Immunosuppressive or cytotoxic drugs.

Please contact the protocol team at actg.teamp1088@fstrf.org if treatment with any of these medications is necessary for clinical care.

4.7 Enrollment Procedures

Prior to implementation of this protocol, and any subsequent full version amendments, each site must have the protocol and the protocol informed consent form(s) approved, as appropriate, by their local institutional review board (IRB)/ethics committee (EC) and any other applicable regulatory entity (RE). Upon receiving final approval, sites will submit all required protocol registration documents to the DAIDS Protocol Registration Office (DAIDS PRO) at the Regulatory Compliance Center (RCC). The DAIDS PRO will review the submitted protocol registration packet to ensure that all of the required documents have been received.

Site-specific informed consent forms (ICFs) WILL be reviewed and approved by the DAIDS PRO and sites will receive an Initial Registration Notification from the DAIDS PRO that indicates successful completion of the protocol registration

process. A copy of the Initial Registration Notification should be retained in the site's regulatory files.

Upon receiving final IRB/EC and any other applicable RE approval(s) for an amendment, sites should implement the amendment immediately. Sites are required to submit an amendment registration packet to the DAIDS PRO at the RCC. The DAIDS PRO will review the submitted protocol registration packet to ensure that all the required documents have been received. Site-specific ICF(s) *WILL NOT* be reviewed and approved by the DAIDS PRO and sites will receive an Amendment Registration Notification when the DAIDS PRO receives a complete registration packet. A copy of the Amendment Registration Notification should be retained in the site's regulatory files.

For additional information on the protocol registration process and specific documents required for initial and amendment registrations, refer to the current version of the DAIDS Protocol Registration Manual.

There will be an enrollment cap for this protocol. Instructions will be provided on the P1088 protocol specific website (http://impaact.group.org). The study team, with approval of the IMPAACT leadership and DAIDS, may change or remove the enrollment cap as needed.

4.8 Co-enrollment Procedures

Co-enrollment is encouraged, except for protocols that would violate the exclusion criteria. All co-enrollments into therapeutic studies require the assent of the protocol chairs of IMPAACT P1088 and co-enrolling protocols.

5.0 STUDY TREATMENT

All eligible subjects will receive the Novartis **Influenza A (H1N1) 2009 monovalent vaccine** at Day 0. If eligible for Step II, subjects will receive vaccine Dose #2 at 21 days (+7 day window) after Dose #1.

5.1 Drug Regimens and Administration

5.11 Regimen

STEP I (DAY 0): Dose #1 – Novartis **Influenza A (H1N1) 2009 monovalent vaccine** 30 mcg administered as TWO-0.5mL (15mcg) injections intramuscularly

STEP II (DAY 21 [+ 7 day window]: Dose #2 – Novartis **Influenza A (H1N1) 2009 monovalent vaccine** 30 mcg administered as TWO-0.5 mL (15 mcg) injections intramuscularly

Note: Any woman that becomes pregnant between Dose #1and Dose #2 will not receive Dose #2 at Day 21 as previously scheduled. Instead, Dose #2 will be deferred until ≥14 weeks gestation, and with the permission of the subjects obstetrician.

Subjects must be registered to Step II through the SDAC/DMC randomization system. A new prescription with the new SID number must be written for the pharmacist to dispense the second dose of the study vaccine.

5.12 Preparation and Administration

The Novartis **Influenza A (H1N1) 2009 monovalent vaccine** will be provided in pre-filled syringes.

Note: If another product becomes available that requires additional preparation (e.g., single-dose vials) prior to administration or if the pre-filled syringes are no longer available, refer to the table below for injection site and needle length guidelines.

Injection Site and Needle Size		
**Intramuscular (IM) injection**<br>Use a 22-25 gauge needle. Choose the injection site and needle length appropriate to the individual's age and body mass.		
**Age**	**Needle Length**	**Injection Site**
Children & Teens (4-18 years)	5/8" – 1"*	Deltoid muscle of arm
	1" – 1 ¼"	Anterolateral thigh muscle*1
**Adults (19 years or older)**		
Male or Female Less than 130 lbs	5/8" – 1"*	Deltoid muscle of arm
Female 130-200 lbs	1" – 1½"	Deltoid muscle of arm
Male 130-260 lbs		
Female 200+ lbs	1½"	Deltoid muscle of arm
Male 260+ lbs		

NOTE: A 5/8" needle may be used only if skin is stretched tight, subcutaneous tissue is not bunched, and injection is made at a 90-degree angle.
***1 It is recommended that all subjects should have study vaccine administered in the deltoid muscle. Vaccinations with study vaccine that are to be administered in the lateral thigh should follow the instructions in the package insert.**

Two pre-filled syringes of Novartis **Influenza A (H1N1) 2009 monovalent vaccine** 15 mcg/0.5 mL will be required for the 30mcg dose. No reconstitution or dilution is necessary.

Administration

Two-0.5 mL (15 mcg) pre-filled syringes will be used to administer the total 30 mcg dose. Two separate, individual injections will be required. The vaccine should be allowed to reach room temperature shortly before use. Shake the syringe before administering the vaccine.

It is recommended that one pre-filled syringe, 0.5 mL (15 mcg) be injected into one of the subject's deltoid muscle(s) or antero-lateral thigh muscle(s) **(see note in the table above).** In addition, using the second pre-filled syringe another injection of 0.5 mL (15 mcg) will be administered into the other deltoid muscle or antero-lateral thigh muscle. However, if the subject requests that both vaccine injections be given in the same deltoid muscle or same antero-lateral thigh muscle, this is acceptable, provided that the injections are given at least 2 inches apart.

A total of 1 mL (30 mcg), divided into two-0.5 ml (15 mcg) injections (pre-filled syringes), must be administered to complete the total vaccination dose.

This product should not be administered intravenously, subcutaneously, or intradermally.

5.2 Drug Formulation

Novartis Vaccine Pre-filled Syringes

The Novartis **Influenza A (H1N1) 2009 monovalent vaccine** is supplied in a single-dose, pre-filled syringe of 0.5 mL at a concentration of 15 mcg / 0.5 mL. The vaccine is a sterile preparation of purified **2009 H1N1 Influenza A virus** antigen in an isotonic buffer solution. Polymyxin and neomycin are used in the manufacturing process of the vaccine and, therefore, are potential impurities in the finished product.

The Novartis **Influenza A (H1N1) 2009 monovalent vaccine** should be stored at 2° to 8° C (35° to 46°F). Product that has been exposed to freezing or stored differently from these conditions must not be used. For additional information regarding formulation, packaging, and stability please refer to the Investigator's Brochure.

5.3 Dose Modifications

There will be no dose modifications. Subjects who do not receive the Dose #2 will be asked to return for safety assessments and for scheduled blood sample collections for immunogenicity assessments (see section 6.4).

5.4 Drug Supply, Distribution and Pharmacy

This unadjuvanted **Influenza A (H1N1) 2009 monovalent vaccine** is manufactured by Novartis and is being provided by the Biomedical Advanced Research and Development Authority (BARDA).

Study product will be available through the NIAID Clinical Research Products Management Center (CRPMC). The IMPAACT pharmacist can obtain study product for the protocol by following the instructions in the manual "Pharmacy Guidelines and Instructions for DAIDS Clinical Trials Networks" in the section entitled "Study Product Management Responsibilities".

The NIAID CRPMC will not provide antiretroviral therapy (e.g., HAART), seasonal trivalent influenza vaccine, syringes or supplies for administration of vaccines as part of this study.

The IMPAACT pharmacist is required to maintain complete records of all study vaccine received from the NIAID CRPMC and subsequently dispensed. All unopened study vaccine must be returned to the NIAID CRPMC after the study is completed or terminated. The procedures to be followed are given in the manual, "Pharmacy Guidelines and Instructions for DAIDS Clinical Trials Networks" in the section "Study Product Management Responsibilities".

6.0 SUBJECT MANAGEMENT

6.1 Toxicity Management

The Division of AIDS Table for Grading the Severity of Adult and Pediatric Adverse Events, Version 1.0, dated December 2004, **Clarification August 2009,** must be used and is available on the RCC web site (**http://rcc.tech-res.com/safetyandpharmacovigilance/).**

- **ALL grades of** adverse events, whether or not associated with study vaccine, should be recorded on the appropriate CRF **and will be assessed in the safety outcomes.**

- The site investigator must receive permission from the study team (actg.teamp1088@fstrf.org) before giving the second vaccination for the following events:
    - All Grade 3 adverse events
    - All Grade 4 injection site reactions or fever
    - All Grade 4 adverse events that are definitely not and probably not related to study vaccine

- For Grade 4 adverse events, other than a local injection site reaction or fever that are definitely, probably or possibly related to study vaccine, Dose #2 should not be given.

6.2 Subject Management

Subjects will remain in the clinic for at least 30 minutes after each vaccination so that clinic personnel can observe for adverse reactions to the vaccine. Subjects will be contacted by telephone or other methods on Day 2 (± 1 day) and Day 10 (± 3 days) after Dose #1 and then again on Day 2 (± 1 day) after Dose #2 for reactogenicity assessments and interview. Additionally, subjects will be seen on Day 10 (± 3 days) after Dose #2 for reactogenicity assessments and safety monitoring (see Appendix IA and IB).

NOTE: A clinic visit is required within 24 hours for any new onset of weakness of legs, tingling of hands and/or feet, or difficulty walking.

In addition, all subjects except women who become pregnant after Dose #1 but prior to Dose #2 (see Appendix IA & IB) will be seen and evaluated 7 months after Dose #1; data will be collected for any signs and symptoms related to Guillain-Barré Syndrome, and other serious adverse events.

The Guillain-Barré Syndrome monitoring tool questionnaire to be completed at the clinic may be accessed by going to the www.fstrf.org website and clicking on the QUALITY OF LIFE link. The form is labeled ‘GBS Baseline and Monitoring Tool – II’.

Subjects should be asked to return to clinic for an unscheduled clinic visit, within 24 hours of development of symptoms consistent with Guillain-Barré Syndrome, such as new onset of weakness of legs, tingling of hands and/or feet, or difficulty walking (see section 6.6).

Subjects will also be asked to return to clinic within 72 hours for assessment for any of the following:

- Assessment of grade 3 or 4 possibly vaccine associated events
- Suspected serious adverse events (other than Guillain-Barré Syndrome),
- Symptoms consistent with influenza-like illness (ILI) (see section 6.5)

It is anticipated that vaccine-associated AEs will occur frequently, but that these will be minor local reactions and side effects that will rarely necessitate interruption of the vaccination schedule. Management of adverse experiences will be according to the best clinical practice and the judgment of the site investigator.

For this protocol, the following definition of H1N1 infection will be used: "Symptoms of influenza-like illness as well as a proven history, by RT-PCR, of novel influenza H1N1 infection, or, has positive influenza diagnostic testing since June 2009 (specificity to H1N1 not required) prior to study entry."

If a subject experiences documented H1N1 infection, as defined in Section 4.23, between Step I and Step II of the protocol, the subject should NOT receive the second dose of vaccine but should continue to be seen at the scheduled study visits, and have blood collected, as per the Schedule of Evaluations.

Antipyretics should not be routinely given in anticipation of adverse events after vaccination, but should not be withheld if symptoms occur. Antipyretics should be recorded when given.

6.3 Criteria for Deferral of Second Dose of Vaccine

If a subject meets any of the criteria described below, Dose #2 should be deferred. If deferred, the subject will have a 7 day window to receive Dose #2. If this period elapses, the site must obtain approval from the study team prior to administering Dose #2 (actg.teamp1088@fstrf.org). Deferral of Dose #2 due to pregnancy is outlined in section 6.7.

6.31 Presence of signs or symptoms that could confound or confuse assessment of vaccine reactogenicity.

6.32 Body temperature ≥ 100.0°F, determined within 24 hours prior to vaccination.

6.33 Presence of a serious, acute infection in the seven days prior to scheduled vaccination.

6.34 Treatment with systemic (oral or parenteral) prednisone or prednisone-equivalents (≥ 2 mg/kg or ≥ 20 mg total dose for ≥ 3 days) within 30 days prior to planned administration of Dose #2.

6.35 Woman becomes pregnant after Dose #1 (see section 6.7)

6.4. Criteria for Permanent Exclusion from Second Dose of Vaccine

If a subject meets any of the exclusion criteria described in section 4.4, Dose #2 cannot be administered. If a subject is excluded from receiving Dose #2 of **Influenza A (H1N1) 2009 monovalent vaccine**, site staff should make all reasonable attempts to request the subject return to clinic for the 7 month visit (end of study). Every attempt should be made to maintain phone contact, to monitor for safety, throughout the study period.

6.5 Evaluation of Influenza-Like Illness (ILI)

Subjects who develop respiratory complaints including sore throat, coryza (runny nose) and/or cough in association with fever $\geq 100.0^{o}F$ will be asked to come to clinic within 72 hours for an unscheduled study visit (see Appendices IA and IB). At this visit, a respiratory specimen will be collected even if fever is by report and not documented. Nasopharyngeal swabs or washes, or tracheal aspirates (for an intubated subject) are acceptable. An assay for **2009 H1N1 Influenza A virus** using RT-PCR will be performed at the core immunology laboratory in batches. Therefore, while the result will be shared with the site team once available, clinicians may opt to obtain additional specimens locally for cultures or PCR for evaluation of ILI.

For this protocol, the following definition of H1N1 infection will be used: "Symptoms of influenza-like illness as well as a proven history, by RT-PCR, of novel influenza H1N1 infection, or, has positive influenza diagnostic testing since June 2009 (specificity to H1N1 not required) prior to study entry."

If a subject experiences documented H1N1 infection, as defined above and in Section 4.23, between Step I and Step II of the protocol, the subject should NOT receive the second dose of vaccine but should continue to be seen at the scheduled study visits, and have blood collected, as per the Schedule of Evaluations.

6.6 Evaluation of Possible Guillain-Barré Syndrome

Subjects who develop any of the following symptoms will be requested to return to clinic within 24 hours for an unscheduled study visit to assess for Guillain-Barré Syndrome ('Unscheduled visit' in Appendices IA and IB):

- new onset of weakness of legs,
- tingling of hands and/or feet,
- difficulty walking

6.7 Management of Pregnancy on Study

HIV-1 infected women who are pregnant should be treated according to the Public Health Service Task Force Recommendations for Use of Antiretroviral Drugs in Pregnant HIV-

Infected Women for Maternal Health and Interventions to Reduce Perinatal HIV Transmission in the United States: Tables - April 29, 2009 (http://www.aidsinfo.nih.gov/Guidelines/)

NOTE: The protocol team encourages the clinical sites to prospectively register the subject's pregnancy in the Antiretroviral Pregnancy Registry. http://www.apregistry.com/reg.htm (In US: 1-800-258-4263).

6.71 Women who become pregnant after receipt of Dose #1 but before Dose #2
Women who have received Dose #1 and are found to be pregnant prior to the Dose #2, will have entry into STEP II delayed until ≥14 weeks of gestation and with permission of their obstetrician. These women will also have an additional clinic visit within 28 days post delivery, as will their infant. Given the increased morbidity and mortality of pandemic H1N1 influenza in pregnant women, completion of the vaccine series is important to their health and, potentially, the health of their infant. These women will be followed as described in Appendix IB.

6.72 Women who become pregnant after Dose #2.
Women who become pregnant after Dose #2 will remain on study with study evaluations as described in Appendix IA.

6.73 Termination of Pregnancy
If a pregnancy is terminated, either spontaneously or electively, the site must notify the P1088 team for instructions (actg.teamp1088@fstrf.org)

6.8 Permanent Study Discontinuation

Criteria for discontinuation from the study are as follows:

- Loss to follow up.
- Subject withdrawal of consent.
- The investigator determines that further participation would be detrimental to the subject's health or well-being.
- The subject fails to comply with the study requirements so as to cause them self harm.

This study may be terminated for safety concerns of the PI, FDA, OHRP, DAIDS, DMID, SMC, participating Institutional Review Boards or Ethics Committees, and other governmental agencies.

7.0 EXPEDITED ADVERSE EVENT REPORTING

7.1 Expedited Adverse Event Reporting to DAIDS

The adverse events (AEs) that must be reported in an expedited fashion to DAIDS Regulatory Compliance Center (RCC) Safety Office include all serious adverse events (SAEs) as defined by ICH guidelines regardless of relationship to the study agent(s). A Serious Adverse Event (SAE) is any untoward medical occurrence that at any dose:

- Results in death
- Is life-threatening[1]
- Requires inpatient hospitalization or prolongation of existing hospitalization[2]
- Results in persistent or significant disability/incapacity, or
- Is a congenital anomaly/birth defect

Important medical events that may not be immediately life-threatening or result in death or hospitalization but may jeopardize the patient or may require intervention to prevent one of the outcomes listed in the definition above must also be reported in an expedited timeframe to DAIDS. Such determination may be made through medical or scientific judgment [ICH E2A]

In addition to reporting all SAEs as defined above, other events that sites must report in an expedited fashion include:

- Symptoms of Guillain-Barre Syndrome such as tingling of hands and/or feet, weakness of legs and new onset of difficulty walking

For all SAE's submitted to RCC, sites must file an updated SAE report to RCC with the final or stable outcome (Status Code p. 5 of the EAE form) unless the SAE reported in the initial EAE form already had a final or stable outcome.

---

[1] The term "life-threatening" refers to an event in which the patient was at risk of death at the time of the event.
[2] Per ICH SAE definition, hospitalization itself is not an adverse event, but is an outcome of the event. Thus, hospitalization in the absence of an adverse event is not regarded as an AE, and is not subject to expedited reporting.
The following types of hospitalization do not require expedited reporting to DAIDS:

- Any admission unrelated to an AE (e.g. for labor/delivery, aging-related cosmetic surgery, administrative admission, or social admission for temporary placement for lack of place to sleep)
- Protocol-specified admission (e.g. for procedure required by protocol)
- Admission for diagnosis or therapy of a condition that existed before receipt of study agent(s) (unless it is a worsening or increasing in frequency as judged by the clinical investigator)

Note: A new AIDS-defining event in a subject already known to be HIV-infected would be considered a worsening of a pre-existing condition (HIV infection).

The study agents for which relationship assessments are required are **Novartis Influenza A (H1N1) 2009 monovalent vaccine**.

The Division of AIDS Table for Grading the Severity of Adult and Pediatric Adverse Events, Version 1.0, dated December 2004, **Clarification August 2009,** must be used and is available on the RCC web site **(http://rcc.tech-res.com/safetyandpharmacovigilance/).**

The protocol-defined expedited event reporting period for this protocol is the entire study duration for an individual subject (from study enrollment until study completion or discontinuation of the subject from study participation for any reason).
After the end of the protocol-defined reporting period defined above, sites must report clinical events that are serious, unexpected adverse drug reactions if the study site staff becomes aware of the event on a passive basis, i.e., from publicly available information.

The timelines and mechanisms for reporting all the events listed above to the DAIDS Regulatory Compliance Center (RCC) Safety Office are defined in the "Manual for Expedited Reporting of Adverse Events to DAIDS" (DAIDS EAE Manual), dated May 6, 2004. The DAIDS EAE Manual is available on the RCC website: **http://rcc.tech-res.com/safetyandpharmacovigilance/.**

Sites using the DAERS internet-based reporting system for submission of EAEs to DAIDS will follow DAERS processes as outlined in DAERS training information. For questions about DAERS, please contact DAIDS-ES at DAIDS-ESSupport@niaid.nih.gov or from within DAERS application itself.

If the site cannot use DAERS to report an AE on an expedited basis, the AE must be documented on the DAIDS Expedited Adverse Event Reporting Form (EAE Reporting Form) available on the RCC website: **http://rcc.tech-res.com/safetyandpharmacovigilance/** and submitted as specified by the DAIDS EAE Manual. For questions about EAE reporting, please continue to contact the RCC.

8.0 STATISTICAL CONSIDERATIONS

8.1 General Design Issues

This is a Phase II study in children and youth ≥ 4 years to < 25 years old who were infected with HIV-1 via perinatal transmission. This study is designed to investigate the safety, reactogenicity, and immunogenicity of a two dose regimen of Novartis **Influenza A (H1N1) 2009 monovalent vaccine**.

The sample will be stratified into 3 age strata, which may differ with respect to immune response. Moreover, factors such as immune status at the time of first immunization and differences in background exposure to the virus across sites participating in the study

may have an impact on response to the vaccine The study will not have an adequate sample size to provide precise estimates of immune response and adverse events rates for all subgroups which may exhibit differing results. Section 8.6 present confidence intervals around potential subgroups of various sizes with a range of potential results to provide an indication of the precision with which these rates can be estimated.

The study will be monitored intensely by the protocol team, which will review data every other week while subjects are accruing and the vaccine is being administered and at least once a month after the last dose of study vaccine has been administered (See Section 8.5). A Safety Monitoring Committee (SMC) will be convened by IMPAACT to review safety information gathered from all study participants.

8.2 Endpoints and Outcome Measures

8.21 Primary Endpoints:

8.211 Adverse Events of Grade 3 or higher severity, including:
- Abnormal laboratory values, signs and symptoms or diagnoses.
- Solicited local AEs, including pain, tenderness, redness, and swelling post each vaccination.
- Solicited systemic AEs, including feverishness, malaise, body aches (exclusive of the injection site), nausea, and headache post each vaccination.

8.212 Adverse Events of Grade 3 or higher severity attributed to the study vaccine.

8.213 Second vaccine dose withheld, due to adverse reactions attributed to first dose.

8.214 Immunologic response, defined as HAI titer ≥ 1:40, at 21 days after Dose #1 and at 10 days after Dose #2.

8.22 Secondary endpoints

8.221 HAI ≥ 1:40 at 6 months after Dose #2.

8.23 Secondary response variables

8.231 Geometric Mean Antibody Titers (GMT) HAI following: 1) Dose #1 and Dose #2, and 2) at 6 months after Dose #2.

8.232 Cell-mediated immune responses, as measured by B-cell and T-cell ELISPOT values and frequency of cytotoxic T cell lymphocytes specific for **2009 H1N1 Influenza A virus**.

8.233 HAI titers against seasonal influenza viruses containing TIV.

8.234 Cell-mediated immune responses to influenza viruses contained in TIV and other antigens.

8.3 Randomization and Stratification

There will be no randomization. Subjects will be stratified by age (at least 40 subjects per group):

Group 1: ≥ 4 years to < 9 years
Group 2: ≥ 9 years to <18 years
Group 3: ≥ 18 years to < 25 years

8.4 Sample Size and Accrual

Approximately 140 children and youth, aged ≥ 4 years to 25 years old, who were infected with HIV-1 via perinatal transmission will be enrolled into this study. We will enroll at least 40 subjects each from three different age groups: age ≥ 4 years to <9 years of age, ≥9 years to < 18 years of age and ≥18 to < 25 years of age. This sample size is expected to yield at least 120 evaluable* subjects, which will provide reasonably precise estimates of rates of adverse events and immunogenic response. Evaluable subjects are defined as having received two doses of Novartis **Influenza A (H1N1) 2009 monovalent vaccine** 21-28 days apart and had all HAI samples for collected up to and including Day 10 post second dose within the protocol specified windows.

Accrual is anticipated to be 4-6 weeks.

*Evaluable subjects are defined as having received two doses of Novartis **Influenza A (H1N1) 2009 monovalent vaccine** 21-28 days apart and had all HAI samples for collected up to and including Day 10 post Dose #2 within the protocol specified windows.

8.5 Safety Monitoring

It is the responsibility of the Protocol Team to interpret safety data and make decisions regarding adverse events to protect subjects from undue risk. In addition, the IMPAACT Network will appoint a Study Monitoring Committee (SMC) to provide independent reviews to ensure subject safety. The SMC is composed of three clinicians and a statistician independent of the P1088 protocol team and vaccine manufacturer.

It is required that the data needed for the toxicity reports be entered into the database within 48 hours of the time at which the results of the laboratory tests or clinical examinations become available.

Reports compiled by the Data Management Center (DMC) will be reviewed and discussed by the Protocol Team on biweekly conference calls while subjects are accruing and the vaccine is being administered. The Protocol Team will meet via conference call at least once a month after the last dose of study vaccine is given. These reports may also be reviewed by the Study Monitoring Committee, if any of the scenarios described below occur, or if any other conditions warrant such a review.

Adverse events will be monitored throughout the follow-up period. If the protocol team identifies any potentially treatment-related toxicity that may compromise subject safety, it will determine whether further enrollment and vaccinations will be paused. Should this occur, the Study Monitoring Committee will review all relevant data and will determine whether, and under what conditions, the study will be allowed to proceed or will be stopped. **For safety monitoring, a vaccine related SAE is an SAE that is judged to be "definitely", "probably', or "possibly related" to study vaccine.**

Further enrollment and vaccinations will be paused for SMC review and recommendation if any of the following are reported across this study or ongoing NIH studies evaluating the 30mcg dose of the Novartis **Influenza A (H1N1) 2009 monovalent vaccine**.

- Any death occurring within the 10 days following administration of study vaccine that was not the result of trauma or accident.
- Any subject having laryngospasm, bronchospasm, or systemic anaphylaxis within 24 hours of administration of study vaccine.
- Two or more subjects with generalized urticaria associated with product administration within 72 hours of administration of study vaccine.
- Any subject with any necrosis at the injection site.
- Three or more subjects with abscesses or ulcerations, associated with study vaccine administration.
- Any subject with a vaccine-related SAE, as defined by ICH, as mentioned in section 7.0.
- Any subject with acute weakness of limbs and or cranial nerve innervated muscles (description of potential signal of Guillain-Barre Syndrome).

The study will also be halted for SMC review and recommendation if, during the 10 days after each vaccination, any of the following occurs:

- 15% or more (minimum of 2 if less than 20 subjects have been vaccinated) of the subjects enrolled in this study experience the same severe (Grade 3 or higher by

the DAIDS Toxicity Table) vaccine-related local reaction that interferes with daily activities. Dimensions of a local reaction are not included in stopping decisions.

- 15% or more (minimum of 2 if less than 20 subjects have been vaccinated) of the subjects in this study experience the same severe (Grade 3 or higher by the DAIDS Toxicity Table) vaccine-related quantitative systemic reactions.

- 15% or more (minimum of 2 if less than 20 subjects have been vaccinated) of the subjects enrolled in this study experience the same severe (Grade 3 or higher by the DAIDS Toxicity Table) vaccine-related subjective systemic reactions, the severity (grade) of which is corroborated by study personnel.

If the study is stopped for any of the above reasons, the SMC will instruct the protocol team under what conditions the study would stop again for a repeat of the same reason.

8.6 Analyses

8.61 Primary Analyses

8.611 Safety

The proportion of all subjects and of subjects within age strata experiencing grade 3 or grade 4 adverse events following the first and second doses of study vaccine will be presented. The precision with which these sample results estimate the rate of similar adverse events in the population represented by the study sample will be reported in terms of 90% confidence intervals (CI) around the sample proportions. This will provide 95% confidence that the rate of severe adverse effects is not greater than the upper limit of the CI around the proportion of subjects with grade 3+ events in the study sample.

Table 1 illustrates the confidence intervals for a range of hypothetical proportions of adverse events. This table shows (e.g.) that a sample finding of no grade 3+ adverse events in a total evaluable sample of 120 subjects would provide 95% confidence that the rate in the population from which the sample was drawn is no greater than 2%. A similar finding within a subsample of 20 subjects would provide 95% confidence that the population rate was no greater than 14%.

Table 1: Exact 90% Confidence Intervals around Potential Proportions of Subjects Exhibiting Grade 3+ Adverse Events (Total Sample and Selected Subsample Sizes)

N	Samples Rate	Lower Limit	Upper Limit
120	0%	0%	2%
	5%	2%	10%
	10%	6%	16%
	25%	19%	32%
	50%	42%	58%
40	0%	0%	7%
	5%	1%	15%
	10%	3%	21%
	25%	14%	39%
	50%	36%	64%
20	0%	0%	14%
	5%	0%	22%
	10%	2%	28%
	25%	10%	46%
	50%	30%	70%

Table 2 shows the probability of detecting at least 1 adverse event of a given type and/or level of severity in the study sample, given a range of hypothesized rates in the population from which the sample was drawn. The table demonstrates that the probability of observing one or more events in a sample of 120 subjects is $\geq$91% with events whose population probabilities are at least 2%, while the probability of observing two or more events is $\geq$88% for events whose population probabilities are at least 3%.

Table 2: Probability of detecting Adverse Events

True Event Rate in the Population Represented by the Study Sample	Probability of Observing Event(s) in the Study Sample (N = 120)	
	1 or more Event	2 or more Events
0.5%	45%	12%
1%	70%	34%
2%	91%	69%
3%	97%	88%
4%	99%	96%
5%	99.8%	98%

8.612 Immunogenicity

The proportion of subjects meeting the criteria for immunologic response following the first and second vaccine doses will be presented. The precision with which this sample result estimates the rate of immunologic response rates in the population represented by the study sample will be reported in terms of 90% CIs around the sample proportions. This will provide 95% confidence that the rate of

immune response is not less than the lower limit of the proportion of study subjects meeting immune response criteria.

Table 3 illustrates the confidence intervals for a range of hypothetical proportions of immunologic response. This table shows (e.g.) that a sample finding of 90 responders in the total sample of 120 subjects (75%) would provide 95% confidence that the rate in the population from which the sample was drawn was no lower than 68%. If a subsample of 20 subjects exhibited a 75% response rate, this would provide 95% confidence that the population rate was no lower than 54%.

Table 3: Exact 90% Confidence Intervals around Potential Proportions of Subjects Meeting Immunologic Response Criteria (Total Sample and Selected Subsample Sizes)

N	Sample rate	Lower Limit	Upper Limit
120	25%	19%	32%
	50%	42%	58%
	75%	68%	81%
	90%	84%	94%
	95%	90%	98%
40	25%	14%	39%
	50%	36%	64%
	75%	61%	86%
	90%	79%	97%
	95%	85%	99%
20	25%	10%	46%
	50%	30%	70%
	75%	54%	90%
	90%	72%	98%
	95%	78%	99.7%

8.62 Key Secondary Analyses

Prediction of immune response will be addressed in secondary analyses. Since this is a new vaccine about which there is considerable uncertainty with respect to the distribution of antibody titers which it may elicit, alternative outcome variables will be explored. These include:

1) The log (10) HAI titer,
2) HAI titer >10, considered indicative of an immune response; and
3) HAI titer >40, which is considered to be likely to be protective.

The distribution of log (10) HAI titer will be examined to determine whether it satisfies the normality assumption required for linear regression analysis. If this is not true, then this method will be not be used. Since the other potential outcome

variables listed above are categorical, the method of analysis for those outcome variables will be logistic regression.

Factors predicting immune response will be examined on data collected after the first vaccination and, again, after the second. A primary predictor will be the age stratification factor. If immune response were to vary significantly across age strata, this would counter indicate pooling across age strata when presenting results. Additional potential predictors will include variables such as: CD4 count or percentage at baseline, viral load at baseline, HAI titers to the seasonal influenza vaccine, ARV treatment, and timing of seasonal influenza vaccine Additional predictors may be explored as additional data concerning the H1N1 virus and the study vaccine become available.

Descriptive analyses examining cell-mediated immunity will make use of ELISPOT data reflecting B and T cell responses to the Novartis **Influenza A (H1N1) 2009 monovalent vaccine**, TIV and other pathogens and cytotoxic T cell lymphocyte (CTL) frequencies against **2009 H1N1 Influenza A virus**. The distributions of baseline ELISPOT values will be examined, and summary statistics including medians and interquartile ranges will be presented. The numbers of spot forming cells measured after the first and second doses of vaccine will be compared to the baseline. The method of analysis will depend upon the distributions of the ELISPOT data and will consist of paired t-tests, if normality assumptions are met, and Wilcoxon Matched Pairs Signed Ranks tests, if they are not. The persistence of memory B cells will be evaluated on specimens collected 6 months after Dose #2. Change from baseline will be evaluated by paired t-tests (or the Wilcoxon test if the data are not normally distributed) to determine whether a significant difference persists. Correlations between B and T cell ELISPOT values will be computed, along with correlations between these values and antibody titers. These will consist of Pearson correlations, if the data are normally distributed or Spearman correlations if normality assumptions are not met. The CTL analyses will consist of descriptive analyses at baseline and after each dose of vaccine. Comparison of CTL frequencies across the 3 time points will be made to determine whether the vaccine increases the percentage of CTL against H1N1 virus that have homing markers for the lung. As with the ELISPOT data, the method of analysis will be paired t-tests or Wilcoxon tests, depending upon whether normality assumptions are met.

9.0 HUMAN SUBJECTS

9.1 Institutional Review Board and Informed Consent

This protocol, the informed consent document (Appendix III), and any subsequent modifications must be reviewed and approved by the IRB or EC responsible for oversight

of the study. Written informed consent must be obtained from the subject (or parents or legal guardians of subjects who cannot consent for themselves, such as those below the legal age). The subject's assent must also be obtained if he or she is able to understand the nature, significance, and risks of the study. The informed consent will describe the purpose of the study, the procedures to be followed, and the risks and benefits of participation. A copy of the consent form will be given to the subject (or parent or legal guardian).

Each site which receives US HHS funding and follows the United States Code of Federal Regulations Title 45-Public Welfare, Part 46-Protection of Human Subjects (also known as the Common Rule) should have on record at the site a plan that detects and addresses any change in guardianship occurring in pediatric subjects and determines when a study subject must have a consent process which involves a legally authorized representative (LAR) other than a family member with guardianship. The plan will include how the site determines when a LAR is initially or no longer needed and how frequently the LAR re-signs the consent. The plan should follow all IRB/EC, local, state and national guidelines. Confirmation of such a plan at a site should be submitted with protocol registration materials.

9.2 Subject Confidentiality

All research laboratory specimens, evaluation forms, reports, and other records will be identified only by a coded number to maintain subject confidentiality. All records will be kept in a secured area. All computer entry and networking programs will be done with coded numbers only. Clinical information will not be released without written permission of the subject, except as necessary for monitoring by FDA, Office for Human Research Protections (OHRP), the NIAID, the local IRB or Ethics Committee.

9.3 Risks and Benefits of the Study

This protocol would most likely be categorized as greater than minimal risk but presenting the prospect of direct benefit to individual subjects (45 CFR 46.405).

9.31 Potential Risks

Clinical trials to evaluate Novartis and other manufacturers, unadjuvanted and adjuvanted **Influenza A (H1N1) 2009 monovalent vaccine** have been initiated. Due to the similarity of the starting material, manufacturing product and composition of the final container product, it is expected that the adverse event (AE) profile of inactivated **Influenza A (H1N1) 2009 monovalent vaccine** will be similar to similar licensed influenza vaccines. Occasionally, adult recipients of influenza vaccines may develop reactions such as fever, body aches, headache, malaise, myalgia, and/or nausea. These may occur more frequently in people who are given the higher dose level of vaccine These reactions are usually greatest

within the first 24 hours after vaccination and last 1 to 2 days. Some subjects may develop reactions at the site of vaccination (redness, swelling, pain, or tenderness). Analgesics (e.g., acetaminophen) and rest will generally relieve or moderate these symptoms. These reactions should go away in 1 to 4 days and should not require additional treatment.

Acute and potentially life-threatening allergic reactions are also possible. Very rarely, occurring in about 1 in 4 million people given a vaccination, there can be a serious allergic reaction to a vaccine. These reactions can cause skin rash (hives), difficulty breathing, swelling around the mouth, throat, or eyes, a fast pulse, sweating, or loss of blood pressure. If these reactions occur they can usually be stopped by giving emergency medications. As with any vaccine or medication, there is a very small chance of a fatal reaction, although researchers do not expect this to occur.

Guillain-Barré syndrome (GBS) is an acute inflammatory neuropathy characterized by weakness, hyporeflexia or areflexia, and elevated protein concentrations in cerebrospinal fluid. Most persons who develop Guillain-Barré syndrome recover completely, although the recovery period may be as little as a few weeks or as long as a few years. About 30% of those with Guillain-Barré syndrome still have residual weakness after 3 years and about 3% may suffer a relapse of muscle weakness and tingling sensations many years after the initial attack. The rate of Guillain-Barré syndrome was significantly increased in individuals receiving the 1976 Swine Influenza (H1N1) vaccine at about 1 per 100,000 vaccine recipients. This has not been seen consistently with other influenza vaccines. Typically onset was within 6 weeks after vaccination. Interestingly, although vaccination rates have increased in the last 10 years, the numbers of reported cases of vaccine-associated Guillain-Barré syndrome have declined. It is unknown if the currently produced Novartis and other manufacturers, unadjuvanted and adjuvanted, **Influenza A (H1N1) 2009 monovalent vaccine** will result in the incidence of Guillain-Barré syndrome that was seen with the 1976 vaccine product as the mechanism leading to this response has not been completely elucidated.

There may be other unknown risks of participation. The risk to the fetus/infant is unknown because animal reproduction studies with the vaccine have not been conducted. There may be other unknown side effects.

9.32 Known Potential Benefits

There are no proven benefits attributable to the receipt of this investigational vaccine, but there is the prospect of benefit. It is possible that vaccination with the Novartis **Influenza A (H1N1) 2009 monovalent vaccine** will result in some protection against infection caused by the H1N1 virus, and may or may not

provide protection against a serious infection with H1N1 influenza, should the virus be contracted. The duration of any such protection is currently unknown. If a woman in this study becomes pregnant after having received vaccination, there may be protection from H1N1 influenza illness in the infant from passive antibody transfer. The dosage of vaccine and schedule of immunization have been selected because in the judgment of the protocol team, they provide the greatest chance of providing protection from **2009 H1N1 Influenza A virus** with the least toxicity. However, the Novartis **Influenza A (H1N1) 2009 monovalent vaccine** is not expected to offer protection against circulating seasonal influenza.

9.4 Study Discontinuation

The study may be discontinued at any time by the NIAID, OHRP, the FDA, SMC, the IRB or ECs or other governmental agencies as part of their duties to ensure that research subjects are protected.

10.0 PUBLICATION OF RESEARCH FINDINGS

Publication of the results of this trial will be governed by IMPAACT policies. Any presentation, abstract, or manuscript will be made available for review by the pharmaceutical sponsors prior to submission.

11.0 BIOHAZARD CONTAINMENT

As the transmission of HIV and other bloodborne pathogens can occur through contact with contaminated needles, blood, and blood products, appropriate blood and secretion precautions will be employed by all personnel in the drawing of blood and shipping and handling of all specimens for this study, as currently recommended by the Centers for Disease Control and Prevention.

See ACTN Guidelines for Shipment and Receipt of Category B Biological Substance shipment document for additional information at the link below:

http://www.hanc.info/labs/Pages/ACTGIMPAACTLabManual.aspx

All infectious specimens will be sent using the ISS-1 SAF-T-PAK mandated by the International Air Transport Association Dangerous Goods Regulations-Packing Instruction 602. Refer to individual carrier guidelines (e.g., Federal Express or Airborne) for specific instructions.

12.0 REFERENCES

(1) Subbarao K, Klimov A, Katz J et al. Characterization of an avian influenza A (H5N1) virus isolated from a child with a fatal respiratory illness. Science 1998; 279(5349):393-396.

(2) Peiris M, Yuen KY, Leung CW et al. Human infection with influenza H9N2. Lancet 1999; 354(9182):916-917.

(3) Dawood FS, Jain S, Finelli L et al. Emergence of a novel swine-origin influenza A (H1N1) virus in humans. N Engl J Med 2009; 360(25):2605-2615.

(4) Peiris JS, Poon LL, Guan Y. Emergence of a novel swine-origin influenza A virus (S-OIV) H1N1 virus in humans. J Clin Virol 2009; 45(3):169-173.

(5) Oxford JS. Influenza A pandemics of the 20th century with special reference to 1918: virology, pathology and epidemiology. Rev Med Virol 2000; 10(2):119-133.

(6) Gatherer D. The 2009 H1N1 influenza outbreak in its historical context. J Clin Virol 2009; 45(3):174-178.

(7) Patriarca PA, Cox NJ. Influenza pandemic preparedness plan for the United States. J Infect Dis 1997; 176 Suppl 1:S4-S7.

(8) World Health Organization. http://www.carec.org/influenzaa-H1N1-pandemic-declaration.html. 2009.

(9) Couch RB, Kasel JA. Immunity to influenza in man. Annu Rev Microbiol 1983; 37:529-549.

(10) Ennis FA, Mayner RE, Barry DW et al. Correlation of laboratory studies with clinical responses to A/New Jersey influenza vaccines. J Infect Dis 1977; 136 Suppl:S397-S406.

(11) Ruben FL, Jackson GG. A new subunit influenza vaccine: acceptability compared with standard vaccines and effect of dose on antigenicity. J Infect Dis 1972; 125(6):656-664.

(12) Ruben FL, Potter CW, Stuart-Harris CH. Humoral and secretory antibody responses to immunization with low and high dosage split influenza virus vaccine. Arch Virol 1975; 47(2):157-166.

(13) Matzkin H, Nili E. Accidental tenfold overdose of influenza vaccine: a clinical and serological study. Isr J Med Sci 1984; 20(5):411-415.

(14) Palache AM, Beyer WE, Sprenger MJ et al. Antibody response after influenza immunization with various vaccine doses: a double-blind, placebo-controlled, multi-centre, dose-response study in elderly nursing-home residents and young volunteers. Vaccine 1993; 11(1):3-9.

(15) Gross PA, Quinnan GV, Jr., Weksler ME, Gaerlan PF, Denning CR. Immunization of elderly people with high doses of influenza vaccine. J Am Geriatr Soc 1988; 36(3):209-212.

(16) Keitel WA, Couch RB, Cate TR et al. High doses of purified influenza A virus hemagglutinin significantly augment serum and nasal secretion antibody responses in healthy young adults. J Clin Microbiol 1994; 32(10):2468-2473.

(17) Keitel WA, Cate TR, Atmar RL et al. Increasing doses of purified influenza virus hemagglutinin and subvirion vaccines enhance antibody responses in the elderly. Clin Diagn Lab Immunol 1996; 3(5):507-510.

(18) Keitel WA, Atmar RL, Nino D, Cate TR, Couch RB. Increasing doses of an inactivated influenza A/H1N1 vaccine induce increasing levels of cross-reacting antibody to subsequent, antigenically different, variants. J Infect Dis 2008; 198(7):1016-1018.

(19) Keitel WA, Atmar RL, Cate TR et al. Safety of high doses of influenza vaccine and effect on antibody responses in elderly persons. Arch Intern Med 2006; 166(10):1121-1127.

(20) Falsey AR, Treanor JJ, Tornieporth N, Capellan J, Gorse GJ. Randomized, double-blind controlled phase 3 trial comparing the immunogenicity of high-dose and standard-dose influenza vaccine in adults 65 years of age and older. J Infect Dis 2009; 200(2):172-180.

(21) Atmar RL, Keitel WA, Patel SM et al. Safety and immunogenicity of nonadjuvanted and MF59-adjuvanted influenza A/H9N2 vaccine preparations. Clin Infect Dis 2006; 43(9):1135-1142.

(22) Treanor JJ, Wilkinson BE, Masseoud F et al. Safety and immunogenicity of a recombinant hemagglutinin vaccine for H5 influenza in humans. Vaccine 2001; 19(13-14):1732-1737.

(23) Treanor JJ, Campbell JD, Zangwill KM, Rowe T, Wolff M. Safety and immunogenicity of an inactivated subvirion influenza A (H5N1) vaccine. N Engl J Med 2006; 354(13):1343-1351.

(24) Hammitt LL, Li S, Patterson-Bartlett J, et.al. Kinetics o fviral shedding and immune responses to cold-adapted influenza vaccine. 10th International Symposium on Respiratory Viral Infections, Singapore, Thailand, Feb 28-Mar 2, 2008.

(25) Quan FS, Compans RW, Nguyen HH, Kang SM. Induction of heterosubtypic immunity to influenza virus by intranasal immunization. J Virol 2008; 82(3):1350-1359.

(26) Gelinck LB, van den Bemt BJ, Marijt WA et al. Intradermal influenza vaccination in immunocompromised patients is immunogenic and feasible. Vaccine 2009; 27(18):2469-2474.

(27) Holland D, Booy R, De LF et al. Intradermal influenza vaccine administered using a new microinjection system produces superior immunogenicity in elderly adults: a randomized controlled trial. J Infect Dis 2008; 198(5):650-658.

(28) Leroux-Roels I, Vets E, Freese R et al. Seasonal influenza vaccine delivered by intradermal microinjection: A randomized controlled safety and immunogenicity trial in adults. Vaccine 2008; 26(51):6614-6619.

(29) Galli G, Hancock K, Hoschler K et al. Fast rise of broadly cross-reactive antibodies after boosting long-lived human memory B cells primed by an MF59 adjuvanted prepandemic vaccine. Proc Natl Acad Sci U S A 2009; 106(19):7962-7967.

(30) Vesikari T, Pellegrini M, Karvonen A et al. Enhanced Immunogenicity of Seasonal Influenza Vaccines in Young Children Using MF59 Adjuvant. Pediatr Infect Dis J 2009; 28(7):563-571.

(31) Atmar RL, Keitel WA, In Compans R.W., et.al. Adjuvants for pandemic influenza vaccines. Vaccines for Pandemic Influenza. Current Topics in Microbiology. Springer Verlag: Berlin, 2009, in press, 2009.

(32) Centers for Disease Control and Prevention (CDC). Serum cross-reactive antibody response to a novel influenza A (H1N1) virus after vaccination with seasonal influenza vaccine. MMWR Morb Mortal Wkly Rep 2009; 58(19):521-524.

(33) Fine AD, Bridges CB, De Guzman AM et al. Influenza A among patients with human immunodeficiency virus: an outbreak of infection at a residential facility in New York City. Clin Infect Dis 2001; 32(12):1784-1791.

(34) Madhi SA, Ramasamy N, Bessellar TG, Saloojee H, Klugman KP. Lower respiratory tract infections associated with influenza A and B viruses in an area with a high prevalence of pediatric human immunodeficiency type 1 infection. Pediatr Infect Dis J 2002; 21(4):291-297.

(35) Madhi SA, Schoub B, Simmank K, Blackburn N, Klugman KP. Increased burden of respiratory viral associated severe lower respiratory tract infections in children infected with human immunodeficiency virus type-1. J Pediatr 2000; 137(1):78-84.

(36) Mendoza Sanchez MC, Ruiz-Contreras J, Vivanco JL et al. Respiratory virus infections in children with cancer or HIV infection. J Pediatr Hematol Oncol 2006; 28(3):154-159.

(37) Kroon FP, van Dissel JT, de Jong JC, van FR. Antibody response to influenza, tetanus and pneumococcal vaccines in HIV-seropositive individuals in relation to the number of CD4+ lymphocytes. AIDS 1994; 8(4):469-476.

(38) Miotti PG, Nelson KE, Dallabetta GA, Farzadegan H, Margolick J, Clements ML. The influence of HIV infection on antibody responses to a two-dose regimen of influenza vaccine. JAMA 1989; 262(6):779-783.

(39) Chadwick EG, Chang G, Decker MD, Yogev R, Dimichele D, Edwards KM. Serologic response to standard inactivated influenza vaccine in human immunodeficiency virus-infected children. Pediatr Infect Dis J 1994; 13(3):206-211.

(40) King JC, Jr., Treanor J, Fast PE et al. Comparison of the safety, vaccine virus shedding, and immunogenicity of influenza virus vaccine, trivalent, types A and B, live cold-adapted, administered to human immunodeficiency virus (HIV)-infected and non-HIV-infected adults. J Infect Dis 2000; 181(2):725-728.

(41) Levin MJ, Song LY, Fenton T et al. Shedding of live vaccine virus, comparative safety, and influenza-specific antibody responses after administration of live attenuated and inactivated trivalent influenza vaccines to HIV-infected children. Vaccine 2008; 26(33):4210-4217.

(42) Vigano A, Zuccotti GV, Pacei M et al. Humoral and cellular response to influenza vaccine in HIV-infected children with full viroimmunologic response to antiretroviral therapy. J Acquir Immune Defic Syndr 2008; 48(3):289-296.

(43) Atashili J, Kalilani L, Adimora AA. Efficacy and clinical effectiveness of influenza vaccines in HIV-infected individuals: a meta-analysis. BMC Infect Dis 2006; 6:138.

(44) Haber P, DeStefano F, Angulo FJ et al. Guillain-Barre syndrome following influenza vaccination. JAMA 2004; 292(20):2478-2481.

(45) Lasky T, Terracciano GJ, Magder L et al. The Guillain-Barre syndrome and the 1992-1993 and 1993-1994 influenza vaccines. N Engl J Med 1998; 339(25):1797-1802.

(46) Schonberger LB, Bregman DJ, Sullivan-Bolyai JZ et al. Guillain-Barre syndrome following vaccination in the National Influenza Immunization Program, United States, 1976--1977. Am J Epidemiol 1979; 110(2):105-123.

(47) Flynn PM, Wilson RB, Kapogiannis BG et al. Improving Seroresponse to Hepatitis B Vaccination in HIV-Infected Adolescents: ATN 024. International AIDS Society, Capetown, SA, July 19-22, 2009 and 1st International Workshop on HIV in Pediatrics, Capetown, SA, July 17-18, 2009.

(48) Hackett S, Hill L, Patel J et al. Clinical characteristics of paediatric H1N1 admissions in Birmingham, UK. Lancet 2009;(374):605.

APPENDIX IA - SCHEDULE OF EVALUATIONS

		**STEP I**				**STEP II**			**STEP I & STEP II**		
	**Screen**[1]	**Day 0**[1]	**Day 2** (±1 day)	**Day 10** (±3 days)	**Day 21**[16] (+7 days)	**Day 0**[16] (+7 days)	**Day 2** (±1 day)	**Day 10** (±3 days)	**7 months** (±1month)	Unscheduled visit[18]	Early Treatment Discont.[19]
		Entry **Dose #1**	1-3 days post 1st dose	7-13 days post 1st Dose	21-28 days post 1st dose	21-28 days post 1st dose **Dose #2**	1-3 days post 2nd dose	7-13 days post 2nd Dose	6-8 months post 1st dose		
Informed Consent	X										
History[2]	X	X			X	X		X	X	X	X
Physical exam[3]	X	X			X	X		X	X	X	X
Documentation of HIV status	X										
Neurological exam[4]	X				X	X		X	X	X	X
Pregnancy Test[5]	X	X				X[17]				X	
**H1N1 Vaccination**		**X**				**X**					
Follow up subject contact[6]			X	X			X				
Reactogenicity assessment and interview [7]		X	X	X	X	X	X	X		X	X
Chemistries[8]	2ml							2ml			
Complete blood count[9]	1ml				1ml						
Immunophenotyping (CD4/CD8/CD19)[10]	2ml										
HIV-1 RNA PCR[11]	2ml										
PBMC & plasma for CMI & neutralizing \antibodies[12, 13]	11-20ml				11-20ml			11–20ml			11–20ml
Serum for HAI[13, 14]	2ml				2ml			2ml	2ml		2ml
Respiratory specimen[15]										X	
TOTAL BLOOD VOLUMES	20-29ml				14-23ml			15-24ml	2ml		13-22.ml

Footnotes:

1. It is preferred that screening and study entry occur on the same day; however, entry may be delayed up to 7 days after screening, if required.
2. A targeted medical history is required at screening/entry, as well as subsequent clinic visits, and should include information on:
   - Cancer, allergies and pertinent medical history
   - Current and past vaccinations within the 2009-2010 seasonal flu vaccine periods, including to the end of the study.
   - List of ARVs taken within the 28 days prior to enrollment and throughout the study period. All other medications will be collected from 28 days prior to study entry to 10 days post Dose #2.
   - CDC classification for HIV status at screening/entry.
3. Physical exam should include height, weight, vital signs (temperature (any method), heart rate, respirations and blood pressure) and additional examination as directed by intercurrent signs and symptoms. This should be collected prior to each vaccination.
4. A targeted neurological exam (lower extremity strength and DTRs).
5. A urine or blood HCG pregnancy test and negative result is required within 72 hours prior to each vaccination for all girls who are of child-bearing potential. If STEP I Day 0 test is positive, the subject may not be enrolled into the study. If the STEP I Day 21 test is positive, Dose #2 should be deferred until the subject is ≥ 14 weeks gestation (section 6.7).
6. All subjects will be contacted, to inquire about adverse events, obtain the information written on the subject memory aid and to remind the participant to maintain the memory aid. <u>Subject memory aids are to be used solely as a memory aid for the participant and may not be utilized as a source document.</u> As such, it will not be collected by the study team and may be discarded at the next visit. If a subject is seen in clinic, a clinic evaluation may be substituted for the telephone call evaluation. An interval history should also be obtained.
7. Reactogenicity assessments will briefly review the subject's history and provide an assessment of AEs at 30 minutes post vaccination and at all telephone calls. Assessments will include review of:
   - erythema,
   - induration,
   - pain and tenderness at the injection site,
   - fever,
   - fatigue,
   - myalgia (exclusive of the injection site),

   The following clinical evaluations will also be collected by interviewing the subject:
   - Assessment of eligibility prior to Dose #1 and Dose #2
   - All unplanned health care provider visits will be reported on the appropriate CRF.
8. Chemistries (SGOT, SGPT and creatinine ONLY) should be obtained within 30 days prior to administration of Dose #1 to obtain a baseline record. The same chemistries should be repeated at the STEP II Day 10 visit, to determine any liver or renal toxicity related to study vaccine.
9. CBC within 30 days; chart abstraction may be used
10. Immunophenotyping, including CD4, CD8, and CD19, within 30 days prior to study entry. Testing obtained for clinical care may be used if available. If not available, study specific immunophenotyping should be obtained. It may be run in the CLIA certified laboratory, at the clinical site. It does not need to be run at a DAIDS IQA certified laboratory.

11. HIV-1 RNA obtained from medical record within 30 days of study entry. It may be run in the CLIA certified laboratory, at the clinical site. It does not need to be run at a DAIDS VQA certified laboratory.
12. Blood draw volumes for PBMC/plasma for the various age groups should be no more than the following:
    - ≥4 to <6 years of age: 11.0ml
    - ≥6 to <25 years of age: 20.0ml
13. Directions for collection, processing, storage and shipping can be found in the P1088 Manual of Operations (MOP) and the Laboratory Processing Chart (LPC).
14. HAI will be performed for **H1N1 2009 H1N1 Influenza A virus** at all visits and for influenza viruses contained in the 2009-2010 vaccine preparation at the screening and 7 month visits.
15. Respiratory specimens, including nasopharyngeal swab, nasal wash, or tracheal aspirate obtained at an unscheduled sick visit for evaluation of influenza like illness (ILI). Instructions for nasopharyngeal swab collection can be found in the MOP. All respiratory specimens will be processed, stored frozen and batched shipped to University of Colorado laboratory as per P1088 MOP and LPC. Respiratory specimens are not required for unscheduled sick visits if the subject is being assessed for possible AEs or GBS, without ILI.
16. STEP I Day 21 and STEP II Day 0 (Dose #2) visits should occur on the same day. However, if a subject comes in to clinic for Dose #2 and is found to have a reason for deferment of STEP II entry, such as fever, Dose #2 may be deferred for up to 7 days (28 days post Dose #1). Day 21 study evaluations should be completed. If the reason for deferral is ILI, a respiratory specimen should be collected. If Dose #2 is delayed beyond 72 hours, a history, physical, neurologic examination, pregnancy test should be repeated prior to administration of Dose #2.
17. If a female subject has a positive urine or serum pregnancy test (hCG) on STEP I Day 21, enrollment in STEP II should NOT be performed and Dose #2 vaccination should NOT be administered until ≥14 weeks of gestation. (See SOE, Appendix IB.)
18. Unscheduled visits are for assessment of ILI, possible Guillain-Barre syndrome or possible AEs. Subjects should be asked to return to clinic within 24 hours of development of symptoms consistent with Guillain-Barre syndrome; within 72 hours for assessment of possible adverse events other than GBS or for symptoms consistent with ILI.
19. For subjects who choose to prematurely discontinue the study, the early study discontinuation visit should occur.

NOTE:
For insufficient blood draws, priorities are as follows:
1. Serum for HAI for shipping to the University of Colorado, IMPAACT Immunology Laboratory
2. Toxicity labs (Chemistries)
3. CD4/CD8/CD19
4. PBMC's and plasma for shipping to the University of Colorado, IMPAACT Immunology Laboratory

APPENDIX IB

STEP II-SCHEDULE OF EVALUATIONS FOR WOMEN WHO BECOME PREGNANT BETWEEN DOSE #1 and DOSE #2 of STUDY VACCINE AND THEIR INFANTS

	STEP II					
	**Day 0 Entry DOSE #2**	**Day 2** Post 2nd Dose	**Day 10** Post 2nd Dose	**Post Delivery**	Unscheduled visit[14]	Early Treatment Discontinuation[15]
	≥14 weeks gestation but prior to delivery	±1 day	± 3 days	Birth + 28 days		
**MATERNAL EVALUATIONS**						
History[1]	X		X	X	X	X
Physical exam[2]	X		X	X	X	X
Neurological exam[3]	X		X	X	X	X
H1N1 Vaccination[4]	**X**					
Fetal Heart Rate	X					
Follow up subject contact[5]		X				
Reactogenicity assessment and interview [6]	X	X	X		X	X
Respiratory specimen[7]					X	
Chemistries[8]	2 ml		2ml			
Complete blood count[9]	X					
PBMC & plasma for CMI & neutralizing antibodies[10]	20 ml		20 ml			20ml
Serum for HAI[10]	2 ml		2ml			2ml
TOTAL BLOOD VOLUMES	24 ml		24 ml			22 ml
**INFANT EVALUATIONS**						
History[11]				X		
Physical exam[12]				X		
Neurological exam[13]				X		

Footnotes:

1. A targeted medical history is required at STEP II entry, as well as subsequent clinic visits, and should include information on:
   - Cancer, allergies and pertinent medical history
   - Current and past vaccinations within the 2009-2010 seasonal flu vaccine periods, including to the end of the study.
   - List of ARVs taken throughout the study period. All other medications will be collected from 28 days prior to study entry to 10 days post Dose #2.
   - Pregnancy outcome information

2. Physical exam should include height, weight, vital signs (oral temperature, heart rate, respirations and blood pressure) and additional examination as directed by intercurrent signs and symptoms. This should be collected prior to vaccination.
3. A targeted neurological exam (lower extremity strength and DTRs).
4. Pregnant women may only receive Dose #2 between ≥14 weeks gestation and prior to delivery, with the permission of her obstetrician. A fetal heart rate will be performed before vaccination and after the reactogenicity exam post vaccination.
5. All subjects will be contacted to inquire about adverse events, obtain the information written on the subject memory aid and to remind the participant to maintain the memory aid. <u>Subject memory aids are to be used solely as a memory aid for the participant and may not be utilized as a source document.</u> As such, it will not be collected by the study team and may be discarded at the next visit.
6. Reactogenicity assessments will briefly review the subject's history and provide an assessment of AEs at 30 minutes post vaccination and at all telephone calls. Assessments will include review of:
   - erythema,
   - induration,
   - pain and tenderness at the injection site,
   - fever,
   - fatigue,
   - myalgia (exclusive of the injection site),

   The following clinical evaluations will also be collected by interviewing the subject:
   - Assessment of eligibility prior to Dose #2
   - All unplanned health care provider visits will be reported on the appropriate CRF.
7. Respiratory specimens should also be obtained at an unscheduled sick visit for evaluation of influenza-like illness (ILI). Specimens should be shipped to University of Colorado laboratory. Respiratory specimens are not required for unscheduled sick visits if the subject is being assessed for possible AEs or GBS, without ILI.
8. Chemistries (SGOT, SGPT and creatinine ONLY) should be obtained prior to administration of Dose #2. The same chemistries should be repeated at Day 10 post Dose #2 to determine any liver or renal toxicity related to study vaccine.
9. CBC within 30 days; chart abstraction may be used
10. Directions for collection, processing, storage and shipping can be found in the P1088 Manual of Operation (MOP)
11. A targeted medical history includes birth and neonatal history including
    - Congenital anomalies
    - List of all medications taken since birth
12. Physical exam should include height, weight, vital signs (temperature, heart rate, and respirations).
13. Neonatal neurological exam
14. Unscheduled visits are for assessment of ILI, possible Guillain-Barre syndrome (GBS) and possible AEs. Subjects should be asked to return to clinic within 24 hours of development of symptoms consistent with GBS; and within 72 hours for assessment of possible adverse events (other than GBS) and for symptoms consistent with influenza-like illness (ILI).
15. Subjects who prematurely discontinue study prior to delivery of their infant require the early treatment discontinuation visit.

APPENDIX II

Public Readiness and Emergency Preparedness Act (PREP act).

This protocol and the vaccine tested are covered under the Public Readiness and Emergency Preparedness act (PREP act). Under the PREP Act, covered persons are immune from liability actions brought from the administration or use of a covered countermeasure that is the subject of a declaration.

On June 15, 2009, HHS secretary Kathleen Sebelius had issued an amendment to the Declaration for use of the PREP act to include the H1N1 vaccines and any associated adjuvants (Federal Register, Volume 74, Number 121, Pages: 30294-30297). The PREP act provides immunity for covered persons (such as Manufacturers, Distributers, Program planners and other Qualified persons who prescribe, administer or dispense the vaccine) from tort liability, unless the injury was caused by willful misconduct.

The PREP Act also authorized a "Covered Countermeasures Process Fund" to provide compensation to eligible individuals who suffer specified injuries from administration or use of a countermeasure pursuant to the declaration. Any requests for compensation must be filed within one year of administration or use of the countermeasure. Requests would go to the HRSA Preparedness Countermeasures Injury Compensation Program (http://www.hrsa.gov/countermeasurescomp/default.htm). Compensation may then be available for medical benefits, lost wages and death benefits to eligible individuals for specified injuries in accordance with regulations published by the Secretary. Eligibility for compensation and the injuries for which compensation may be available are further defined by regulation.

An individual who suffers a serious physical injury or death from administration and use of the vaccine must first seek compensation from the Covered Countermeasures Process Fund. A serious physical injury means an injury that is life threatening, results in, or requires medical or surgical intervention to prevent, permanent impairment of a body function or permanent damage to body structure. Any compensation will be reduced by public or private insurance or worker's compensation available to the injured individual.

If no funds have been appropriated to the compensation program, the Secretary does not make a final determination on the individual's request within 240 days, or if the individual decides not to accept the compensation, the injured individual or his representative may pursue a tort claim in the United States District Court for the District of Columbia, but only if the claim involves willful misconduct, is pled with particularity required under the PREP Act, verified, and accompanied by an affidavit by a physician who did not treat the individual and certified medical records. Any award is reduced by any public or private insurance or worker's compensation available to the injured individual. Awards for non-economic damages, such as pain, suffering, physical impairment, mental anguish, and loss of consortium are also limited. If the individual accepts compensation, or if there is no willful misconduct, the individual does not have a tort claim that can be filed in a United States Federal or a State court.

APPENDIX III

DIVISION OF AIDS
INTERNATIONAL MATERNAL PEDIATRIC ADOLESCENT AIDS
CLINICAL TRIALS GROUP (IMPAACT)

SAMPLE INFORMED CONSENT

P1088: "A Phase II Study to Assess the Safety and Immunogenicity of an Inactivated **Influenza A (H1N1) 2009 Monovalent Vaccine** in HIV-1 Perinatally Infected Children and Youth"
Version 2.0, dated April 23, 2010

SHORT TITLE FOR THE IMPAACT P1088 STUDY: Safety of an H1N1 Influenza Vaccine in HIV-1 Perinatally Infected Children and Youth.

'You' as used in this consent form, refers to you and/or your adolescent or child (if he/she is a minor requiring consent).

INTRODUCTION

You are being asked to take part in this research study because you have perinatally-acquired human immunodeficiency virus (HIV), the virus that causes AIDS. Perinatally-acquired means that you became infected with HIV around the time of your birth. This study is sponsored by the National Institutes of Health (NIH). The doctor in charge of this study at this site is: (insert name of Principal Investigator). Before you decide if you want to be a part of this study, we want you to know about the study.

This is a consent form. It gives you information about this study. The study staff will talk with you about this information. You are free to ask questions about this study at any time. If you agree to take part in this study, you will be asked to sign this consent form. You will get a copy to keep.

WHY IS THIS STUDY BEING DONE?
H1N1 virus, historically known as 'swine flu virus', is a new strain of influenza (flu) virus that causes illness in people. H1N1 influenza virus may cause serious, sometimes life-threatening illness in some healthy people. This illness can result in severe pneumonia, which may require hospitalization.

Recent reports by the Centers for Disease Control and Prevention (CDC) show that children and youth are more likely to have severe illness and need hospitalization from H1N1 influenza virus compared with adults.

The purpose of this research study is to determine if this vaccine which has been licensed by the United States Food and Drug Administration (FDA) is safe and will help the body's normal defenses against **the effects of** H1N1 influenza in HIV-1 infected children and youth. The **licensed**

vaccine to be used in this study may not prevent influenza virus infection, but may prevent severe disease and possibly death which has been seen with this H1N1 influenza virus in children.

This vaccine is produced by Novartis, a pharmaceutical company. The vaccine is "inactivated", which means virus used to make the vaccine has been killed, and cannot cause influenza. This vaccine **contains a trace amount of thimerosal (mercury).** The vaccine is made with products from eggs. **If you are allergic to egg protein (eggs or egg products), you would not be able to receive this vaccine.** The vaccine also contains small amounts of the antibiotics neomycin and polymyxin. If you are allergic to one of these antibiotics, you would not be able to receive this vaccine.

The "immune response" is how your body recognizes and defends itself against bacteria, viruses, and substances that may be harmful to the body. There is no information on the safety or immune response to this vaccine in children with HIV. We want to see if your body will produce antibodies (proteins that fight infection) that will prevent or fight H1N1 influenza virus infection; this is one type of "immune response".

The study will look at:
- The safety of the vaccine (after each of two doses). This will be done by observing you following the vaccine shots, and asking you about any side effects you may notice after each shot.
- Your ability to form antibodies in response to the vaccine shots. This will be done by taking your blood at certain time points.
- How long your antibodies stay in your blood. This will be done by doing a blood test on you at time points up to 7 months after the 1st dose of H1N1 influenza vaccine.
- Your immune response to the vaccine if you also get the seasonal influenza vaccine.
- Your CD4 levels (your immune response to HIV) and blood HIV levels (viral load).

WHAT DO I HAVE TO DO AS PART OF THIS STUDY?
If you agree to take part in this study, you will be asked questions to be sure you can participate in this study.

At the screening visit, we will ask about your family history, including Guillain-Barré Syndrome. Guillain-Barré Syndrome is a very rare but serious disorder affecting the nervous system that is explained in more detail later in this consent. In addition, you will be asked for permission to review your medical record.

At the screening visit and at 4 scheduled visits during the next 7 months, you will be asked to return to clinic for the following procedures:
- Study staff will ask you some questions about your medical history, including immunization, and use of any past or present anti-HIV drugs.
- Study staff will perform a physical exam (height and weight, blood pressure, temperature, pulse and respirations) and a neurological exam (testing for reflexes in your joints, how well you can balance, walk and sit).

- About 10-14 teaspoons of blood (depending on your age) will be drawn over the entire study for basic tests (chemistries, CD4 count) as well as special laboratory tests (to test your immune response to the flu virus vaccine).
- Girls/women who have had their first menstrual period must have a pregnancy test. A small amount of urine or blood (less than 1 teaspoon) will be taken for this test. You will be informed of the test result as soon as it is available. If you are found to be pregnant at study entry, then you will not be able to participate in this study. However, study personnel can give you information about a study of this vaccine in pregnant women, if you are interested. If you are having sex that could lead to pregnancy, you or your partner must agree to use at least one method of birth control to avoid pregnancy while you are on this study (see "Are There Risks Related to Pregnancy" below).
- These visits will last approximately 30 to 45 minutes.
- On your first or second visit to clinic, you will be given two injections of the study vaccine.
- You will receive a second set of injections approximately 21 days later.

In addition to these visits, a member of the study staff will call you or see you in clinic, up to three times during the time you are in this study to ask you about any reactions you may have had to the study vaccine.

You will receive two doses of vaccine approximately 21 days apart. The vaccine will be given by injection with a needle in a muscle in the upper arm or in the thigh if the participant is a child. For each dose of vaccine, you will receive 2 injections. After vaccine administration, you will remain in the clinic for at least 30 minutes (½ hour) to make sure that no reactions to the vaccine develop. You will need to report any unusual or serious reactions that occur after you leave the clinic. You will be given a diary as a memory aid so that you can write down symptoms or unusual reactions that occur.

If you have any new weakness of legs, tingling of hands and/or feet, or new difficulty walking after you have been vaccinated, you will be asked to come back to clinic within 24 hours. If you have other side effects or flu-like symptoms after either of the vaccine doses, you may be asked to come back to the clinic within 72 hours.

<u>Sick visits</u>
At any time during the course of the study, if you have a fever of $\geq 100^{o}$F or any signs or symptoms of an upper respiratory illness such as cough, runny nose, sore throat, you will be asked to come into the clinic within 72 hours of your symptoms developing. A wipe of your nasal passages with a cotton swab or a washing from the nose to test for influenza will be done from this sample. This visit will take about ½ hour to complete.

The results of this test will not be available during the study. If you have any of these symptoms after your first dose of vaccine, the 2nd dose of vaccine may be delayed.

Off-Treatment Study Visits (Follow-up Period)
If you are unable to receive the second dose of vaccine, you will be asked to continue with the remaining study visits.

Early Study Discontinuation Visit
If you no longer want to be in this study, or no longer can be in this study, you will be asked to come back to the clinic one last time. At this last visit, some or all the laboratory tests described before will be done if tests have not been done at a recent study visit. About 1 ½ to 3 ½ teaspoons of blood will be drawn for these tests. No vaccine will be given at this study visit. You will have a physical exam and review of symptoms and medications at this visit. This visit will take about ½ hour to complete.

OTHER INFORMATION
When the study is over, results will be available from your study site. You will be given the results of your routine laboratory tests when they become available. The results of special laboratory tests (to test your immune response to the flu virus) will not be provided to you or your doctor. These tests will not be run at the time they are received. Instead they will be run in batches or at the end of the study. Any blood samples that are not used after 3 years following the end of the study analysis will be destroyed.

HOW MANY PEOPLE WILL TAKE PART IN THIS STUDY?
About 140 HIV-1 perinatally infected children and youth will take part in this study.

HOW LONG WILL I BE IN THIS STUDY?
You will be in this study for 7 months.

WHY WOULD THE DOCTOR TAKE ME OFF THIS STUDY EARLY?
The study doctor may need to take you off the study early without your permission if:

- The study is cancelled by the U.S. Food and Drug Administration (FDA), National Institutes of Health (NIH), the Office for Human Research Protections (OHRP), or the site's Institutional Review Board (IRB). An IRB is a committee that watches over the safety and rights of research subjects.
- A Study Monitoring Committee (SMC) recommends that the study be stopped early. An SMC is an outside group of experts that monitors the study.
- You are not able to attend the study visits as required by the study.

The study doctor may also need to take you off the study vaccine without your permission if:

- Continuing the study vaccine may be harmful to you
- You need a treatment that you may not take while on the study

If you must stop receiving the study vaccine before the study is over, the study doctor may ask you to continue to be part of the study and return for some study visits and procedures.

WHAT WILL HAPPEN IF I BECOME PREGNANT WHILE ON THE STUDY?

- If you become pregnant after receiving the first vaccination but before the second vaccine:
  - The second vaccine will be delayed until after you have been pregnant for at least 14 weeks. This will delay exposure of the unborn child to this investigational vaccine until after the most critical stage of development.
  - You will receive the second vaccination at this time but only if your obstetrician agrees.
  - The study staff will call you approximately 2 days after your second vaccination to ask you about any reactions you may have had after the study vaccine.
  - You will be asked to come to the clinic for a visit at about 10 days after the second vaccination. At this visit, you will have about 4½ teaspoons of blood drawn and we will also perform a physical and neurological exam, and ask you some questions.
  - You and your baby will be asked to return to the clinic to have a history, physical and neurological exam within 28 days after your delivery.

- If you become pregnant after receiving both study vaccinations, we will continue to follow you as described earlier.

WHAT ARE THE RISKS OF THE STUDY?

Risk of Blood Draws

You may feel faint or may feel some discomfort while having blood taken. There may be some swelling, bleeding, or bruising where the needle goes into the skin, or a small blood clot may develop. There is a small risk of infection forming where the needle goes into the skin to take blood.

Risks Related to the Vaccine

The dose of study drug is higher than the recommended dose for healthy children because children with HIV infection may not respond as well as healthy children to the lower dose of vaccine.

There is no information on side effects with this specific H1N1 vaccine but this vaccine is similar to other influenza vaccines. This vaccine is similar to that used in other influenza vaccines. This is one of the vaccines being prepared for general use in all people in the United States. However, it is made from a new virus, so at this time, there is limited information on the safety of the vaccine.

Life threatening allergic reactions to vaccines are very rare. If they do occur, it is usually within a few minutes to a few hours after the vaccination. Side effects that might be expected from the H1N1 vaccine would include fever as well as skin redness, pain, tenderness, soreness and swelling at the site of injection. Any information on side effect or risks that is learned from H1N1 vaccination on other groups of people will be provided to you as quickly as possible.

Occasionally, there can be serious allergic reactions to a vaccine. These reactions can cause any of the following symptoms:

- Skin rash (hives)
- Fever
- Nausea
- Vomiting
- Diarrhea
- Abdominal pain
- Cough
- Sore throat
- Achiness
- Feeling tired (fatigue)
- General feeling of illness
- **Myalgia (muscle aches)**

Serious Vaccine Related Reactions:

- Difficulty breathing
- Swelling around the mouth, throat, or eyes
- Fast pulse
- Sweating

If these reactions occur they can usually be stopped by the study staff giving emergency medications. These symptoms usually occur soon after having the vaccine. As with any vaccine or medication, there is a very small chance of a fatal reaction, although researchers do not expect this to occur.

Guillain-Barré Syndrome (GBS), a very rare but serious illness affecting the nervous system that makes people very weak, is not usually seen following routine influenza vaccination. However, some cases of Guillain-Barré were reported after large numbers of people received the 1976 "swine flu" vaccine. It is unknown if the H1N1 vaccine will cause Guillain-Barré syndrome. The study staff will assess you/your child for symptoms of Guillain-Barré at each clinic visit and any phone call.

<u>About Guillain-Barré Syndrome</u>

The peripheral nerves send sensory information (like pain or temperature) from the body to the brain, and motor (movement) signals from the brain to the body. In Guillain-Barré, the body's immune system attacks these nerves. Symptoms of Guillain-Barré include weakness and numbness or tingling in the legs and arms, and possible loss of movement and feeling in the legs, arms, upper body, and face. Complete recovery from Guillain-Barré can take anywhere from a few months to a few years. Although most people recover completely from Guillain-Barré, some do not. The frequency of Guillain-Barré is about 1 to 2 cases in every 100,000 people per year in the United States. It strikes men and women, young and old equally.

ARE THERE RISKS RELATED TO PREGNANCY?

This section applies to you if you can become pregnant. It is not known if the vaccine used in this study can harm unborn babies. If you are having sex that can lead to pregnancy, you or your partner must agree to use one of the methods of birth control listed below as long as you are in this study. You may discuss these choices with the study staff.

- Hormonal birth control drugs that prevent pregnancy given by pills, shots or placed on or under the skin
- Male or female condoms with or without a cream or gel that kills sperm.
- Diaphragm or cervical cap with a cream, or gel that kills sperm.
- Intrauterine device (IUD)

Condoms are recommended because their appropriate use is the only contraception method effective for preventing HIV-1 transmission.

If you can become pregnant, you must have a pregnancy test before you enter the study. The test must be negative. If you think you may be pregnant at any time during the study, you should tell your study staff right away. This study does not provide any treatment or care for pregnancy but the study staff will talk to you about your choices.

ARE THERE BENEFITS TO TAKING PART IN THIS STUDY?

If you agree to take part in this study, there may be a direct benefit to you, but no guarantee can be made. You may develop antibodies against H1N1 influenza virus infection which may prevent infection, or lessen your symptoms if you get infected. It is also possible that you may receive no benefit from being in this study. Information learned from this study may help others who have HIV.

WHAT OTHER CHOICES DO I HAVE BESIDES THIS STUDY?

Alternatives to your participation in this study include receiving from your health care provider the seasonal flu vaccine and **Influenza A (H1N1) 2009 Monovalent Vaccine. Alternatively, you may choose not to receive any vaccine.** Please talk to your health care provider about these and other choices available to you.

WHAT HAPPENS IF I AM INJURED?

If you suffer physical injury from this study, the study doctor will provide immediate medical treatment. The study doctor will also provide referrals to appropriate health care facilities. The cost for this treatment will be charged to your insurance company. There is no program for compensation either through this institution or the National Institutes of Health (NIH). No financial compensation by the doctors that gave you the vaccine will be made for any discomfort suffered because of participation in this study. You will not be giving up any of your legal rights by signing this consent form.

This vaccine and the clinical trial are covered by the Public Readiness and Emergency Preparedness (PREP) Act which limits your ability to sue if you/your child develop a reaction to the vaccine. A Federal program has been created to help pay for medical care and other specific expenses of people who have serious reactions that are caused by the vaccine. To be eligible for this program, you must

file a claim for yourself within one year of the vaccination. The program is administrated by the Health Resources and Services Administration. An information sheet about the PREP Act, and the Federal program, including how to file a claim will be provided to you.

WHAT ABOUT CONFIDENTIALITY?

To help us protect your privacy, we have obtained a Certificate of Confidentiality from the National Institutes of Health. With this Certificate, the researchers cannot be forced to disclose information that may identify you/your child, even by a court subpoena, in any federal, state, or local civil, criminal, administrative, legislative, or other proceedings. The researchers will use the Certificate to resist any demands for information that would identify you, except as explained below. The Certificate cannot be used to resist a demand for information from personnel of the United States Government that is used for auditing or evaluation of federally funded projects or for information that must be disclosed in order to meet the requirements of the federal Food and Drug Administration (FDA).

People who may review your records include the U.S. Food and Drug Administration, Office for Human Research Protections (OHRP), the site IRB (insert name of site IRB), the National Institutes of Health, study staff, study monitors, and their designees.

You should understand that a Certificate of Confidentiality does not prevent you from voluntarily releasing information about your participation in this research. If an insurer, employer, or other person obtains your written consent to receive your research information, then the researchers may not use the Certificate of Confidentiality to withhold that information.

The Certificate of Confidentiality does not prevent the researchers from disclosing voluntarily, without your consent, information that would identify you as a participant in the research project under the following circumstances: possible child abuse and/or neglect or risk of harm to you, your child, or others.

WHAT ARE THE COSTS TO ME?

Taking part in this study may lead to added costs to your insurance company. In some cases it is possible that your insurance company will not pay for these costs because you are taking part in a research study.

WHAT ARE MY RIGHTS AS A RESEARCH SUBJECT?

Taking part in this study is completely voluntary. You may choose not to be part of this study. You may stop taking part in this study at any time. You will be treated the same no matter what you decide.

We will tell you about new information from this or other studies that may affect your health, welfare or willingness to stay in this study. If you want to know the results of the study once it is completed, you should let the study staff know.

WHAT SHOULD I DO IF I HAVE QUESTIONS OR PROBLEMS?

For questions about this study or a research-related injury, contact:

- name of the investigator or other study staff
- telephone number of above

For questions about your rights as a research subject, contact:

- name or title of person on the Institutional Review Board (IRB) or other organization appropriate for the site
- telephone number of above

SIGNATURE PAGE

If you have read this consent form (or had it explained to you), all your questions have been answered and you agree to take part in this study or to allow your child to take part in this study, please sign your name below.

____________________	__________________________________
Participant's Name (print)	Participant's Signature and Date
__________________________	__________________________________
Participant's Legal Guardian (print)<br>(As appropriate)	Legal Guardian's Signature and Date
______________________	__________________________________
Study Staff Conducting<br>Consent Discussion (print)	Study Staff Signature and Date
