# Supplementary material for: Characterization of Functional Antibody and Memory B-Cell Responses to pH1N1 Monovalent Vaccine in HIV-Infected Children and Youth
Source: PLoS One. 2015 Mar 18;10(3):e0118567. doi: 10.1371/journal.pone.0118567 (PMC4364897; doi:10.1371/journal.pone.0118567)
Supplement: S2 Protocol — (DOCX) [file pone.0118567.s003.docx]

**New Works Concept Sheet 114:**

**Title:** Functionality and Determinants of Antibody Responses to H1N1 pandemic (pdm) Vaccine in HIV-Infected Children and Adolescents

**Chairs:** Adriana Weinberg

The overarching goal of this proposal is to provide an in-depth characterization of the immune responses to H1N1pdm vaccine of HIV-infected children and to increase our understanding of the immune defects that lead to decreased immunogenicity of vaccines in this population.

**Aim 1. To determine the functionality of antibody responses to H1N1pdm vaccine in HIV-infected children and adolescents.**

HAI assays measure antibodies that bind to the viral hemagglutinin without discriminating functional from non-functional antibodies. The goal of this aim is to determine if the antibodies that are made by HIV-infected children in response to H1N1pdm vaccines are protective against wild-type infection and if the HAI titers of these individuals reflect functional antibodies. We will measure the viral neutralizing antibody titers generated in response to vaccination and evaluate their correlation with HAI titers in 90 children enrolled in P1088, equally distributed across age groups, selected such that they will represent the entire spectrum of HAI responses observed in our study.

Microneutralization (microneut) assays will be performed on sera collected at baseline, post-dose 1 and post-dose 2 using methods previously described (Weinberg et al. JAIDS 2010). Microneut data will be analyzed similarly to the HAI data, using titers ≥40 as an indication of protection and 4-fold titer increases as an indication of boosting or response. In addition, correlation analyses of Neut and HAI titers will be performed. The findings will indicate if the antibodies made by HIV-infected children in response to H1N1pdm vaccine are functional and if HAI titers are a surrogate marker of protective responses in this population.

**Aim 2. To determine the B-cell characteristics associated with poor antibody responses of HIV-infected children and adolescents.**

Although HIV does not infect B cells, the virus affects maturation and function of these cells via direct and indirect mechanisms. As HIV also severely decreases CD4 cell numbers and function, it is unclear whether the poor vaccine immunogenicity in HIV-infected individuals is due to B or T cell dysfunction or both. The goal of this aim is to characterize the phenotypic and functional characteristics of B cells that are associated with decreased antibody responses to H1N1pdm vaccine. In each of the 3 IMPAACT H1N1pdm vaccine studies, approximately 25% of the participants did not reach protective antibody titers after immunization, which is in stark contrast with the 1 to 2% failure rate observed in immunocompetent vaccine recipients. The mixture of responders and non-responders will allow us to identify the B cell phenotypic and functional characteristics that correlate with lack of response.

Using our NICHD laboratory funds, we will assess B cell function by ELISPOT as outlined in the original special immunology sections of the parent studies. We will complement the ELISPOT assays of 90 subjects in P1088, described under Aim 1, with a detailed flow cytometric characterization of the B cell response to the vaccine. The proportion of naïve, memory and transitional B cells and their functional markers will be assessed using mAbs anti-CD19, CD20, CD21, CD27, CD10, IgM, BAFF receptor and CXCR5. These assays will use cryopreserved PBMC collected at baseline and after each dose of vaccine, which have already been transferred from the clinical sites to our laboratory.

We anticipate that low antibody responses to the vaccine will positively correlate with high proportions of transitional B cells and/or unswitched memory B cells and/or low levels of BAFF receptor, essential for antibody production and commonly down-regulated in HIV-infected individuals, and/or CXCR5, essential for lymphoid follicular migration where B cell maturation takes place, and frequently down-regulated in HIV-infected individuals. We have data on CD4 cell numbers, plasma HIV RNA levels, age, ethnicity, etc. from the study population and we will explore the effect of these parameters on B cell maturation and function. This aim will considerably increase our understanding of the clinical significance of the B-cell dysfunction of HIV-infected children.

**Aim 3. To determine the effect of vitamin D levels on the immunogenicity of H1N1pdm vaccine in HIV-infected children and adolescents.**

Vitamin D is an important co-factor of the immune response. Previous studies showed that many HIV-infected individuals have low levels of the 1, 25-dihydroxy vitamin D3 active metabolite, which may contribute to their immunodeficiency. Low vitamin D levels were associated with increased incidence of opportunistic infections in HIV-infected individuals and may also affect responses to vaccines. We will measure 1, 25-dihydroxy vitamin D3 levels in the baseline serum samples of 90 P1088 participants, described above, and perform correlation analyses of vitamin D levels with HAI and Neut titers and with B- and T-cell ELISPOT results after each dose of vaccine. We will also control for other variables, such as CD4 cell numbers and plasma HIV RNA concentrations, which typically affect responses of HIV-infected individuals to vaccines. Since vitamin D levels vary across ethnic groups, we will pay special attention to the interactions between antibody levels, ethnicity and vitamin D levels. The findings could readily influence clinical care, since vitamin D supplementation is readily achievable.

**Responsiveness to ARRA**

1. This research will provide financial support to hire a new full-time research assistant.
2. It will also use reagents manufactured in the US.
