# Supplementary material for: Characterization of Functional Antibody and Memory B-Cell Responses to pH1N1 Monovalent Vaccine in HIV-Infected Children and Youth
Source: PLoS One. 2015 Mar 18;10(3):e0118567. doi: 10.1371/journal.pone.0118567 (PMC4364897; doi:10.1371/journal.pone.0118567)
Supplement: S1 List — (DOCX) [file pone.0118567.s004.docx]

Institutional Review Boards of P1088 IMPAACT sites:

St. Jude Children’s Research Hospital Institutional Review Board

The Children's Hospital of Philadelphia Institutional Review Board

University of California San Diego Institutional Review Board

Baylor College of Medicine Institutional Review Board

Bronx-Lebanon Hospital Institutional Review Board

San Juan City Hospital (Puerto Rico) Institutional Review Board

Children's Hospital of Michigan Wayne State University Institutional Review Board

Duke University School of Medicine Institutional Review Board

Columbia University Institutional Review Board

New Jersey Medical School, Rutgers Biomedical and Health Sciences Institutional Review Board

University of California Los Angeles Institutional Review Board

Harbor UCLA Medical Center, Memorial Health Services Research Council Institutional Review Board

University of Colorado Denver, Colorado Multiple Institutional Review Board (COMIRB)

South Florida CDC Ft Lauderdale, Broward Health Institutional Review Board

Rush University Cook County Hospital (Chicago) Institutional Review Board

Miller Children's Hospital Long Beach, Memorial Health Services Research Council Institutional Review Board

University of Maryland Institutional Review Board

Western New England Maternal Pediatric Adolescent AIDS CTU, UMass Institutional Review Board

University of Miami Institutional Review Board

Jacobi Medical Center, Albert Einstein College of Medicine Institutional Review Board

Seattle Children's Hospital Institutional Review Board

SUNY Stony Brook Institutional Review Board

Ann and Robert H. Lurie Children's Hospital of Chicago Institutional Review Board

University of Southern California Health Sciences Institutional Review Board

University of Florida Health Science Center Jacksonville (UFHSCJ) Institutional Review Board

Strong Memorial Hospital, University of Rochester Institutional Review Board

University of California San Francisco Institutional Review Board

University of Alabama at Birmingham Institutional Review Board for Human Use

University of Puerto Rico Medical Sciences Campus Institutional Review Board

Children's National Health System Institutional Review Board

University of South Florida Tampa Institutional Review Board

Boston Children’s Hospital Institutional Review Board

Howard University (Medical) Institutional Review Board

New York University (New York) Institutional Review Board

New York Medical College (Metropolitan Hospital) Institutional Review Board

Johns Hopkins Medicine Institutional Review Board

Boston University/Boston Medical Center Institutional Review Board
