# Supplementary material for: Characterization of Functional Antibody and Memory B-Cell Responses to pH1N1 Monovalent Vaccine in HIV-Infected Children and Youth
Source: PLoS One. 2015 Mar 18;10(3):e0118567. doi: 10.1371/journal.pone.0118567 (PMC4364897; doi:10.1371/journal.pone.0118567)
Supplement: S1 Table — (DOCX) [file pone.0118567.s005.docx]

**Supplemental Table 1. Characteristics of the NWCS 114 Sample compared to the Parent Study Population**

| **Variable** | **Subjects from Parent Study Not Included in NWCS 114 Analysis (N=65)** | **Subjects from Parent Study Included in NWCS 114 Analyses (N=90)** | **Total (N=155)** | **P-Value** |
| --- | --- | --- | --- | --- |
| Gender Female | 29 (45%) | 47 (52%) | 76 (49%) | 0.42* |
| Race and Ethnicity |  |  |  |  |
| Black | 42 (65%) | 53 (59%) | 95 (61%) | 0.38* |
| Latino | 17 (26%) | 33 (37%) | 50 (32%) | 0.17* |
| Age (yrs) |  |  |  |  |
| Mean | 12.49 (5.88) | 13.17 (5.85) | 12.88 (5.86) | 0.48** |
| Median | 11 (7, 18) | 14 (8, 18) | 13 (7, 18) |  |
| ARV regimen, N (%) |  |  |  |  |
| HAART | 57 (88%) | 80 (89%) | 137 (88%) | 0.81* |
| Other | 8 (12%) | 10 (11%) | 18 (12%) |  |
| CD4 Percent |  |  |  |  |
| Mean | 34.35 (8.93) | 32.69 (9.25) | 33.38 (9.13) | 0.27** |
| N | 64 | 90 | 154 |  |
| CD8 Percent |  |  |  |  |
| Mean | 36.46 (12.11) | 37.19 (13.57) | 36.89 (12.95) | 0.73** |
| N | 64 | 90 | 154 |  |
| Log 10 RNA Count |  |  |  |  |
| Mean^a^ | 2.11 (0.77) | 2.10 (0.85) | 2.10 (0.82) | 0.96** |
| N | 62 | 90 | 152 |  |
| There was no statistical difference between the sample of subjects chosen for the NWCS 114 analysis and those who were not included, based on gender, race/ethnicity, ARV regimen, age, CD4%, CD8% or HIV viral load. | | | | |
| *Fisher's Exact Test | | | | |
| **t Test | | | | |
