# Supplementary material for: Characterization of Functional Antibody and Memory B-Cell Responses to pH1N1 Monovalent Vaccine in HIV-Infected Children and Youth
Source: PLoS One. 2015 Mar 18;10(3):e0118567. doi: 10.1371/journal.pone.0118567 (PMC4364897; doi:10.1371/journal.pone.0118567)
Supplement: S2 Table — (DOCX) [file pone.0118567.s006.docx]

**Supplemental Table 2. Correlations of pH1N1 immune responses with HIV disease characteristics CD4% and HIV viral load**

| **Variable 1** | **Variable 2 at baseline** | **Post Dose 1** | |
| --- | --- | --- | --- |
|  |  | **ρ (p value)** | **N** |
| pH1N1 HAI Titers | CD4% | 0.04 (0.73) | 90 |
|  | HIV RNA cp/mL | -0.09 (0.43) | 90 |
| Microneutralization Titers | CD4% | -0.15 (0.16) | 88 |
|  | HIV RNA cp/mL | 0.04 (0.74) | 88 |
| Antibody Avidity (post-dose 2) | CD4% | -0.05 (0.68) | 88 |
|  | HIV RNA cp/mL | -0.07 (0.53) | 88 |
| B-cell memory IgG ASC | CD4% | -0.15 (0.36) | 40 |
|  | HIV RNA cp/mL | -0.12 (0.45) | 40 |
| IFNγ | CD4% | 0.07 (0.55) | 72 |
|  | HIV RNA cp/mL | -0.09 (0.45) | 72 |
| IL-2 | CD4% | -0.19 (0.12) | 72 |
|  | HIV RNA cp/mL | 0.13 (0.29) | 72 |
| There was no significant correlation between pH1N1 vaccine immune response and baseline HIV characteristics of CD4 percent or viral load. | | | |
