# Supplementary material for: Characterization of Functional Antibody and Memory B-Cell Responses to pH1N1 Monovalent Vaccine in HIV-Infected Children and Youth
Source: PLoS One. 2015 Mar 18;10(3):e0118567. doi: 10.1371/journal.pone.0118567 (PMC4364897; doi:10.1371/journal.pone.0118567)
Supplement: S3 Table — (DOCX) [file pone.0118567.s007.docx]

**Supplemental Table 3. Phenotypic Characterization of B-Cell Subsets** **at Baseline and After pH1N1 Vaccination**

| **Variable (%)^a^** | **Baseline** | | **Post-dose 1** | | **Post-dose 2** | |
| --- | --- | --- | --- | --- | --- | --- |
|  | **Median (IQR)** | **N** | **Median (IQR)** | **N** | **Median (IQR)** | **N** |
| CD19+CD21+CD27+ (resting memory) | 31.55 (23.45,42.35) | 56 | 33.70 (26.00,43.70) | 51 | 35.00 (24.00,43.30) | 48 |
| CD19+CD21+CD27-(naïve) | 36.70 (20.75,45.10) | 56 | 35.10 (18.20,48.60) | 51 | 30.65 (18.90,44.80) | 48 |
| CD19+CD21-CD27-CD20+ (tissue-like memory) | 3.06 (2.04,5.07) | 56 | 2.26 (1.61,4.10) | 51 | 2.18 (1.29,5.53) | 48 |
| CD19+CD21-CD27-CD20- (transitional) | 3.86 (2.52,6.76) | 56 | 2.92 (1.12,7.29) | 51 | 4.25 (2.25,8.02) | 48 |
| CD19+CD21-CD27+CD20- (plasmablasts) | 10.48 (6.04,14.34) | 56 | 8.58 (4.49,13.69) | 51 | 8.81 (5.82,15.51) | 48 |
| CD19+CD10+ (immature) | 65.35 (52.70,77.80) | 56 | 69.60 (56.40,81.84) | 51 | 68.40 (56.55,76.53) | 48 |
| CD19+CD10+CD27+ (immature activated) | 34.80 (22.40,51.25) | 56 | 36.00 (25.40,51.30) | 51 | 37.90 (24.80,53.30) | 48 |
| CD19+HLADR+CD38+ (activated) | 15.70 (11.95,25.80) | 48 | 16.70 (11.40,20.70) | 41 | 16.10 (10.60,21.00) | 42 |
| CD19+BAFFR+ | 83.84 (77.75,89.50) | 48 | 83.90 (76.00,90.63) | 41 | 83.05 (76.94,90.20) | 42 |
| CD19+TACI+ | 5.23 (2.46,12.84) | 48 | 7.69 (1.63,15.84) | 41 | 7.02 (1.74,15.47) | 42 |
| CD19+CXCR5+ | 81.30 (76.6,88.14) | 48 | 83.40 (78.9,88.2) | 41 | 84.60 (75.6,89.3) | 42 |
| a. Subsets, measured by flow cytometry, are expressed as a percentage of the parent CD19+ B-cell population. | | | | | | |
| There was no significant change from baseline to post-dose 1 or post-dose 2 for any of the B-cell subsets measured. | | | | | | |
