# Supplementary material for: Characterization of Functional Antibody and Memory B-Cell Responses to pH1N1 Monovalent Vaccine in HIV-Infected Children and Youth
Source: PLoS One. 2015 Mar 18;10(3):e0118567. doi: 10.1371/journal.pone.0118567 (PMC4364897; doi:10.1371/journal.pone.0118567)
Supplement: S4 Table — (DOCX) [file pone.0118567.s008.docx]

**Supplemental Table 4. Correlations between pH1N1 and sH1N1 HAI titers**

| **Time Period** | **N** | **rho** | **P** |
| --- | --- | --- | --- |
| Baseline (week 0) | 78 | 0.08 | 0.46 |
| Post-dose 1 | 78 | 0.27 | **0.02** |
| Post-dose 2 | 77 | 0.24 | **0.03** |
| 7 months post-dose 1 | 79 | 0.16 | 0.15 |
| There was a significant correlation between pandemic H1N1 and seasonal H1N1 titers at post-dose 1 and post-dose 2. No correlation was found pre-vaccination or at 7 months. | | | |
